# Multifunctional Catalyst Combination for the Direct Conversion of CO_2_ to Propane

**DOI:** 10.1021/jacsau.1c00302

**Published:** 2021-09-02

**Authors:** Adrian Ramirez, Pierfrancesco Ticali, Davide Salusso, Tomas Cordero-Lanzac, Samy Ould-Chikh, Christian Ahoba-Sam, Aram L. Bugaev, Elisa Borfecchia, Sara Morandi, Matteo Signorile, Silvia Bordiga, Jorge Gascon, Unni Olsbye

**Affiliations:** † KAUST Catalysis Center (KCC), King Abdullah University of Science and Technology, Thuwal 23955, Saudi Arabia; ‡ Department of Chemistry, NIS Center and INSTM Reference Center, University of Turin, Turin 10125, Italy; § SMN Centre for Materials Science and Nanotechnology, Department of Chemistry, University of Oslo, Oslo N-0315, Norway; ∥ The Smart Materials Research Institute, Southern Federal University, Sladkova 178/24, Rostov-on-Don 344090, Russian Federation

**Keywords:** CO_2_ conversion, hydrogenation, propane, tandem catalysts, zeolites, kinetics, reaction mechanism

## Abstract

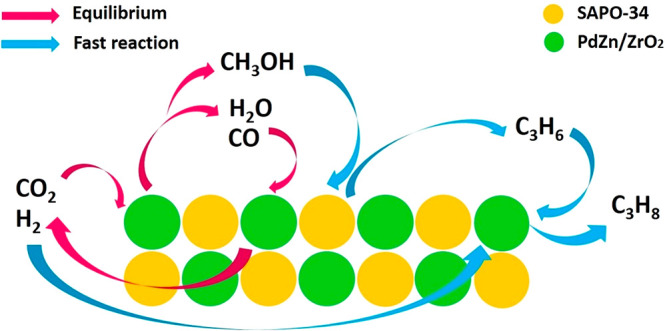

The production of carbon-rich hydrocarbons via CO_2_ valorization is essential for the transition to renewable, non-fossil-fuel-based energy sources. However, most of the recent works in the state of the art are devoted to the formation of olefins and aromatics, ignoring the rest of the hydrocarbon commodities that, like propane, are essential to our economy. Hence, in this work, we have developed a highly active and selective PdZn/ZrO_2_+SAPO-34 multifunctional catalyst for the direct conversion of CO_2_ to propane. Our multifunctional system displays a total selectivity to propane higher than 50% (with 20% CO, 6% C_1_, 13% C_2_, 10% C_4_, and 1% C_5_) and a CO_2_ conversion close to 40% at 350 °C, 50 bar, and 1500 mL g^–1^ h^–1^. We attribute these results to the synergy between the intimately mixed PdZn/ZrO_2_ and SAPO-34 components that shifts the overall reaction equilibrium, boosting CO_2_ conversion and minimizing CO selectivity. Comparison to a PdZn/ZrO_2_+ZSM-5 system showed that propane selectivity is further boosted by the topology of SAPO-34. The presence of Pd in the catalyst drives paraffin production via hydrogenation, with more than 99.9% of the products being saturated hydrocarbons, offering very important advantages for the purification of the products.

## 1 Introduction

Carbon capture and utilization (CCU) is critical to reducing CO_2_ emissions and mitigating global warming.^1^ Through the production of carbon-rich hydrocarbons via CO_2_ valorization,^2,3^ CCU is expected to pave the route for the transition to renewable, non-fossil-fuel-based energy sources.^4^

One of the most promising approaches toward CO_2_ valorization consists of the combination of conventional metallic catalysts with acidic zeolites.^5^ This combination can directly transform CO_2_ to a great variety of chemicals with a selectivity above the limitation of the Anderson–Schulz–Flory distribution (ASF).^6^ However, most of the work to date has been limited to the production of either olefins or aromatics, with the rest of essential hydrocarbons being barely studied.^7^ This is of particular importance as, for a real circular carbon economy (CCE), other common hydrocarbons and fuels should be as well produced via CO_2_.^8^ One example of essential hydrocarbon with high demand is propane.

Propane is nowadays produced as a byproduct in two other processes, natural gas processing and petroleum refining. The propane market is expected to grow at 5% per year, adding up to 350 million metric tons per year in 2025^9^ (with the potential therefore to account for 1050 million metric tons of CO_2_), being the industrial and residential sectors the two most relevant actors. In industrial processes, propane is used for large-scale applications such as furnaces and heaters. And in the residential sector, propane is widely used for air conditioning, heating, refrigeration, production of textiles, lighting, and other uses. However, despite the obvious importance of this hydrocarbon, propane formation from CO_2_ has been barely touched in the available literature,^10,11^ and only a few prior papers targeted paraffin formation, mostly from syngas.^12−17^

Hence, with propane production in mind, in this work we have developed a highly active and selective PdZn/ZrO_2_+SAPO-34 multifunctional catalyst. In particular, we took advantage of the hydrogenation function of the Pd component and the high C_3_ selectivity of the SAPO-34 to develop a catalytic system with high selectivity toward propane. Zn and ZrO_2_ were also included in the formulation because of the well-known ability of these elements to efficiently convert CO_2_ to methanol,^18^ the first step in the overall multifunctional mechanism.^6^

The resulting PdZn/ZrO_2_+SAPO-34 catalytic system displays a total selectivity to propane higher than 50% with a CO_2_ conversion close to 40% and only 20% of CO selectivity at 350 °C, 50 bar, and 1500 mL g^–1^ h^–1^. To the best of our knowledge, this is the highest total selectivity per pass ever reported for a single C_2+_ hydrocarbon during CO_2_ valorization.^11^ We attribute these results to the intimate contact between the PdZn/ZrO_2_ and SAPO-34 components that shifts the overall reaction equilibrium, ultimately boosting CO_2_ conversion and minimizing CO selectivity. Kinetic modeling of the catalytic data alongside with thermodynamic equilibrium calculations fully support this hypothesis. Lastly, the presence of Pd in the catalyst drives paraffin production via hydrogenation, with more than 99.9% of the products being saturated hydrocarbons, offering very important advantages for the purification of the products.

## 2 Experimental Section

### 2.1 Catalyst Preparation

Pd(CH_3_COO)_2_ (>99.9%), Zn(CH_3_COO)_2_ (99.99%), Zr(OH)_4_, PVP (Mwt 10000), DMF (99.8%), and ethylene glycol (99.8%) were purchased from Sigma-Aldrich and used as received. ZSM-5 (SiO_2_/Al_2_O_3_ = 23) was purchased from Zeolyst. SAPO-34 (SiO_2_/Al_2_O_3_ = 0.5) was purchased from ACS materials. All the zeolites were dried at 120 °C for 12 h and calcined at 550 °C for 2 h prior to testing.

The PdZn/ZrO_2_ catalyst was obtained by a colloidal impregnation method. Briefly, 5 g L^–1^ of Pd(CH_3_COO)_2_ was dissolved in DMF and 20 g L^–1^ Zn(CH_3_COO)_2_ dissolved in ethylene glycol were prepared. Eight grams of PVP was added to 80 mL of the Zn precursor solution and heated to 80 °C to obtain a clear yellow solution. Fifty milliliters of the Pd precursor solution was added to the clear yellow zinc/PVP solution amidst stirring and heated to 100 °C under reflux for 2 h. The colloidal mixture was cooled, centrifuged, and washed with acetone and then dispersed in ethanol. The dispersed colloidal mixture in ethanol was added to 6 g of Zr(OH)_4_ powder and stirred for 20 h at room temperature. The resulting mixture was oven-dried and calcined at 500 °C for 3 h. The multifunctional PdZn/ZrO_2_+zeolite catalysts were prepared by mortar mixing of both components with a 1:1 mass ratio.

### 2.2 Catalytic Tests and Kinetic Modeling

Catalytic tests were executed in a 16 channel Flowrence from Avantium. Fifty milligrams of the stand-alone PdZn/ZrO_2_ catalyst and 100 mg of composite catalyst with PdZn/ZrO_2_+zeolite with a mass ratio of 1/1 in a mixed bed configuration were typically used. Both functions were pelletized together in a 1/1 ratio, and then sieved to a particle size 150–250 μm. The mixed feed had 22.5 vol % of CO_2_, 72.5 vol % of H_2_, and 5% of He as internal standard. For the catalyst activity evaluation, we aimed at a gas hourly space velocity (GHSV) value of 12000 mL g^–1^ h^–1^ per channel. One of the 16 channels was always used without catalyst as blank. The reaction temperature was typically set at 350 °C. Prior to feeding the reaction mixture all samples were pretreated in situ with a pure H_2_ atmosphere for 4 h at 400 °C. The tubes were then pressurized to 30 bar using a membrane-based pressure controller. Extra runs were also performed in order to evaluate the kinetics of the reactions using the stand-alone PdZn/ZrO_2_ catalyst and the multifunctional catalytic system. For this purpose, operation conditions were ranged between 250 and 350 °C, 30–50 bar and 1500–30 000 mL g^–1^ h^–1^.

Reaction products were analyzed online in a gas chromatograph. The GC is an Agilent 7890B with three detectors, a TCD and 2 FIDs. TCD is equipped with 2 Haysep precolumn and a MS5A, where He, H_2_, CH_4_, CO_2_, and CO are separated. FIDs are equipped with Gaspro and an Innowax columns. Gaspro separates C_1_–C_8_ hydrocarbons and DME. Innowax separates oxygenates and aromatics.

Conversion (*X*, %) and selectivity (*S*_Cn_, %) are defined as follow:
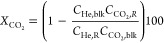
1
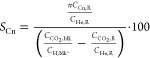
2where *C*_He,blk_, *C*_He,R_, *C*_CO2,blk_, and C_CO2,R_ are the concentrations determined by GC analysis of He in the blank, He in the reactor effluent, CO_2_ in the blank, and CO_2_ in the reactor effluent, respectively, and *C*_Cn,R_ is the concentration of the reactor effluent determined be GC analysis of a product with *n* carbon atoms. The error in carbon balance was better than 2.5% in all cases.

Experimental results collected with both the stand-alone PdZn/ZrO_2_ catalyst and the multifunctional PdZn/ZrO_2_+SAPO-34 catalytic system were fitted by means of kinetic modeling in order to estimate and compare the rates of the reactions. Because of the characteristics of the experimental setup, some assumptions can be made, thereby simplifying the system. More details can be found in the Supporting Information. Briefly, a steady-state plug flow model, working at isothermal and isobaric conditions, is assumed for the reactor. To avoid mathematical uncertainty and strictly compare reaction rates, we considered two equilibrium reactions: CO_2_ hydrogenation to methanol and the reverse water–gas shift reaction. Equilibrium constants were considered with empirical correlations, and the results were contrasted with those obtained from the *FactSage* equilibrium database. For the multifunctional system where methanol is further transformed to hydrocarbons, a fast conversion of methanol to propane is assumed, with both methanol-to-propene and propene hydrogenation considered to be quite faster than CO_2_/CO/MeOH equilibria.

### 2.3 Characterization of Catalysts

The powder X-ray diffraction (XRD) measurements were performed by using a Bruker D8 Discover, with a Cu Kα source and a Lynx Eye silicon detector. The diffractograms were scanned with step size of 0.02° in the 2θ range of 10–90°. The crystalline phase was identified by comparison with data from the inorganic crystal structure database, *ICSD*.

Nitrogen adsorption-desorption isotherms were recorded using a Micromeritics ASAP 2040 at –196 °C. Samples were previously evacuated at 100 °C for 16 h. The Brunauer–Emmett–Teller (BET) method was used to calculate the surface area. The *p*/*p*_0_ range for BET analysis was 0.067 < *p*/*p*_0_ < 0.249.

The temperature-programmed desorption (TPD) experiments were carried out in a Micromeritics ASAP 2020. The catalyst samples were first heated in helium flow at 350 °C for 4 h, followed by cooling to 50 °C. After cooling, the zeolites were saturated in ammonia and the temperature of the samples was increased linearly at a rate of 10 °C/min. Ammonia was fed at atmospheric pressure with a 5% vol. NH_3_ concentration was diluted in Helium. The ammonia desorption was continuously monitored by a thermal conductivity detector.

Thermogravimetric analyses (TGA) of catalysts were carried out in a TGA/DSC1 STAR-e system apparatus (Mettler Toledo). Before TPO experiments, the catalyst was submitted to stripping under a N_2_ stream (50 mL min^–1^) up to the reaction temperature using a heating ramp of 10 °C min^–1^. After that, the sample was cooled and stabilized at 100 °C. The temperature was then increased up to 850 °C using a heating ramp of 5 °C min^–1^ under an air flow of 50 mL min^–1^ to ensure the total combustion of coke.

XAS measurements were performed at the Quick-XAS ROCK^19^ beamline of the French synchrotron SOLEIL. K-edges of Pd and Zn were separately collected in transmission mode. Si(220) and Si(111) monochromators were employed to scan Pd and Zn absorption edges, in the range 23.8–25.7 and 9.3–10.7 keV, respectively. The PdZn/ZrO_2_ catalyst was physically mixed with zeolite ZSM-5 (1:1 weight ratio) and packed in a quartz capillary reactor. To optimize the signal quality in transmission mode, we chose a capillary of Ø 1 mm for the Zn K edge and 2.5 mm for the Pd K edge. The capillary was connected to a gas flow system while heating was provided by a heat gun. The measurement followed the catalyst activation protocol used for the catalytic tests, i.e. XAS spectra were recorded during a heating ramp (RT-400 °C) under H_2_ gas flow (10 mL/min). The reported XAS spectra resulted from the average of 500 and 120 quick-EXAFS spectra (0.5 s/scan). Two ionization chambers were used to measure *I*_0_ and *I*_T_ while a third one measured a reference metal foil used for energy alignment. ZnO and PdO were also measured as reference compounds in the form of self-supporting pellets. XAS data analysis (XANES normalization and energy calibration, χ(*k*) EXAFS extraction, and Fourier transform (FT)-EXAFS calculation) were carried out using the *Athena* software from the *Demeter* package.^20^ The EXAFS fitting of PdZn active phase was performed using the *ARTEMIS* code of the *Demeter* package.^20^ Pd–Pd and Pd–Zn scattering paths were generated by the *FEFF6* code implemented in *ARTEMIS* using starting interatomic distances taken from literature.^21^ The passive amplitude reduction factor was obtained from EXAFS analysis of aPd reference foil. Pd–Pd and Pd–Zn coordination numbers were fixed to the bulk values (4 and 8, respectively), whereas the energy shift (*E*_0_), radial distances (Δ*R*), and Debye–Waller factors (σ^2^) were fit as free variables.

Absorption IR spectra were run using a PerkinElmer FTIR 2000 spectrophotometer equipped with a Hg–Cd–Te cryo-detector, in the range of wavenumbers 7200–580 cm^–1^ at a resolution of 2 cm^–1^. The powder of PdZn/ZrO_2_ was compressed in self-supporting discs (∼20 mg cm^–2^) and placed in quartz IR cells suitable for thermal treatments in controlled atmosphere and spectra recording at room temperature (RT). Moreover, a commercial stainless-steel cell (Aabspec), allowing thermal treatments in situ under vacuum or controlled atmosphere and the simultaneous registration of spectra at temperatures up to 600 °C, was employed. Before IR measurements, catalyst underwent oxidizing or reducing pretreatment: in both cases, it was outgassed at 400 °C for 30 min and then oxidized in dry oxygen (40 mbar) or reduced in hydrogen (40 mbar) at 400 °C for 30 min. Reduction treatment simulates the reduction step performed prior to the catalytic tests. Prereduced catalyst will be named activated catalyst. First of all, interaction with the reagents involved in the CO_2_-to-methanol process, i.e., H_2_ and CO_2_, was investigated. In particular, interaction with H_2_ (10 mbar) was performed in situ at increasing temperature on the preoxidized catalyst to study the effect of the activation step used for the catalytic tests. Interaction with CO_2_ (20 mbar) was studied at RT on both preoxidized and activated catalyst. To characterize the supported metal phase, we carried out CO adsorption for increasing pressure up to 20 mbar at RT on the activated catalyst.

Transmission electron microscopy (TEM) of the samples was performed with a Cs-probe corrected Titan microscope from Thermo Fisher Scientific by operating it at an accelerating voltage of 300 kV and with a beam current of 0.5–0.8 nA. Dark-field imaging was performed by scanning TEM (STEM) coupled to a high-angle annular dark-field (HAADF) detector. The STEM-HAADF data were acquired with a convergence angle of 17.1 mrad and a HAADF inner angle of 50 mrad. Furthermore, a X-ray energy-dispersive spectrometer (FEI SuperX, ∼0.7 sR collection angle) was also utilized in conjunction with DF-STEM imaging to acquire STEM-EDS spectrum-imaging data sets (image size: 512 × 512 pixels, dwell time 4 μs). During the acquisition of these data sets, at every image-pixel, a corresponding EDS spectrum was also acquired for generating simultaneously the elemental maps of Si, Al, Pd, Zn, Zr, and O atoms. It is also pertinent to note herein that spectrum-imaging data sets were acquired in the so-called frame mode, in which the electron beam was allowed to dwell at each pixel for only a time of few microseconds in order to keep a total frame time to 2 s or less. Both imaging and spectroscopy data sets for each sample were acquired as well as analyzed with a newly developed software package called *Velox* from Thermo Fisher Scientific. The elemental maps for Si, Al, Pd, Zn, Zr, and O atoms were computed using the extracted intensity of their respective Kα lines after background subtraction. The generated maps were slightly postfiltered by applying a Gaussian filter (sigma = 0.5). Because of the possible air sensitivity of the PdZn/ZnO solid after H_2_ activation and CO_2_ hydrogenation, the sample was handled inside an Ar-filled glovebox. The specimen was prepared by simply shaking a small amount of dry powder and the TEM grid inside a 2 mL sample vial. The TEM grid was retrieved and mounted in a Gatan double-tilt vacuum transfer TEM holder, model 648 that was used for the transfer into the microscope.

UV-Raman spectra were collected with a Renishaw inVia Raman spectrometer, adopting a Coherent MotoFred 300C frequency doubled Ar^+^ laser emitting at 244 nm as excitation source. The spectrometer is equipped with a 15× objective, a 3600 lines/mm grating and a Peltier cooled CCD detector. To prevent sample decomposition, we kept samples under movement during the measurements with a specifically designed setup.^22^

## 3 Results and Discussion

### 3.1 Catalytic Tests for the CO_2_ Hydrogenation to Propane

**Figure 1:**
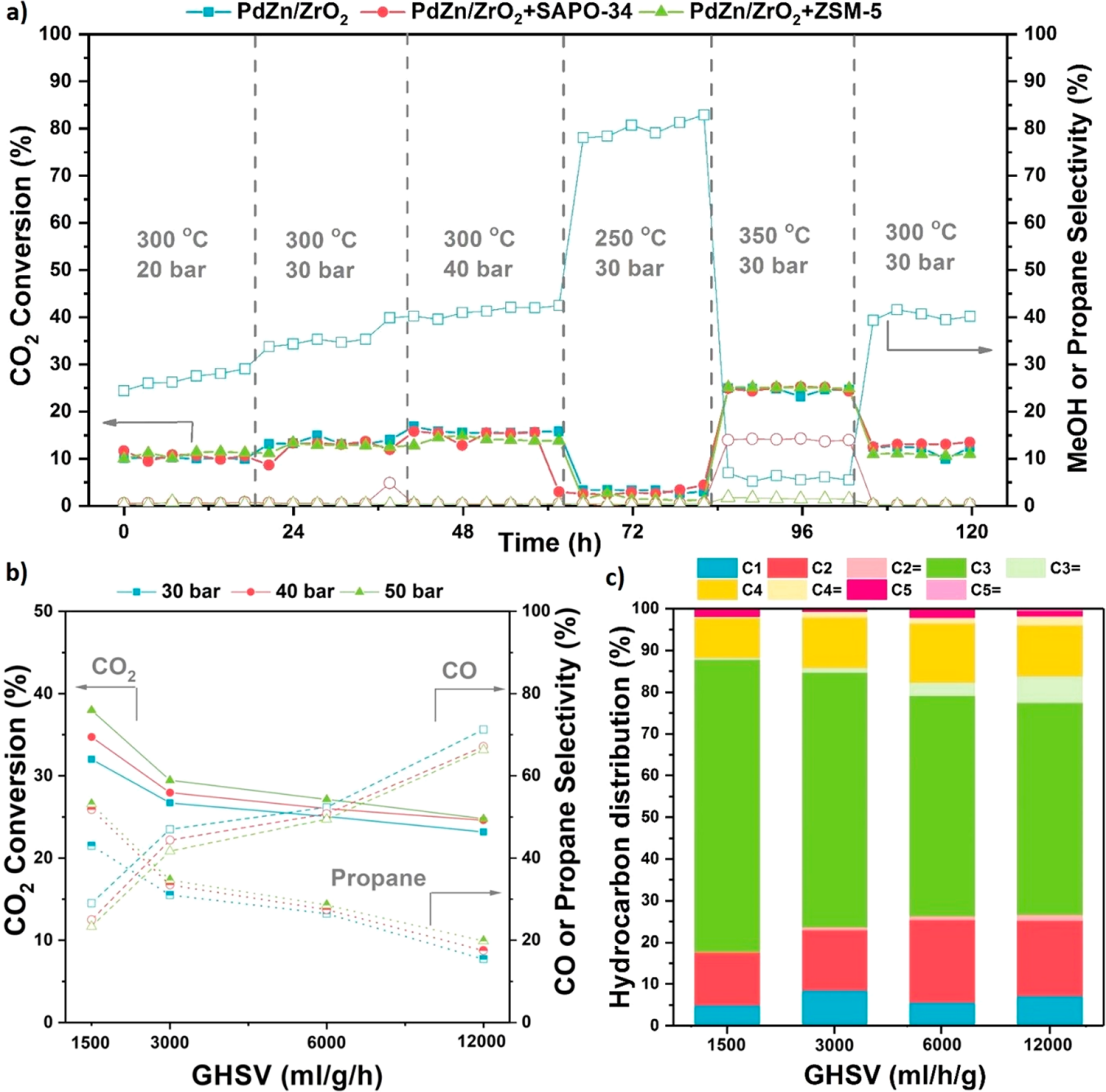
(a) CO_2_ conversion (filled symbols) and MeOH (for PdZn/ZrO_2_) or propane (for PdZn/ZrO_2_+SAPO-34 and PdZn/ZrO_2_+ZSM-5) selectivity (empty symbols) at several screening conditions. H_2_/CO_2_ = 3, 12 000 mL g^–1^ h^–1^. (b) CO_2_ conversion (filled symbols) and CO (empty symbols) or propane (half empty symbols) selectivity for the PdZn/ZrO_2_+SAPO-34 system at different space velocities and pressures. 350 °C, H_2_/CO_2_ = 3. MeOH selectivity was lower than 1% at all conditions. (c) Detailed hydrocarbon distribution (CO free) of the PdZn/ZrO_2_+SAPO-34 combined system for the CO_2_ conversion to hydrocarbons at different space velocities. CO_2_:H_2_ 1:3, 350 °C, 50 bar.

We first performed a catalytic screening over the stand-alone PdZn/ZrO_2_ catalysts and their combination with SAPO-34 and ZSM-5, the two most common zeolites employed in multifunctional catalysts for CO_2_ conversion.^7^ These control experiments were focused on monitoring MeOH selectivity for the stand-alone PdZn/ZrO_2_ catalysts and that of propane for the multifunctional systems. In particular, the effect of reaction pressure (20, 30, and 40 bar) and temperature (250 °C, 300 and 350 °C) was evaluated. The results are summarized in Figure 1a. CO_2_ conversion is similar for the three systems (filled symbols, left axis) and it increases with pressure and temperature, in agreement with the process thermodynamics.^11^ On the other hand, selectivity follows a completely different trend (empty symbols, right axis). For the stand-alone PdZn/ZrO_2_ catalysts, MeOH selectivity also follows the process thermodynamics, increasing with pressure and decreasing with temperature. A full picture of the allowed equilibrium selectivities (lines) and the obtained experimental data (dots) can be observed in Figure S1. Note that the orange lines indicate the maximum allowed methanol selectivity considering the CO_2_ to methanol equilibrium. For the multifunctional systems, however, no propane is observed for temperatures lower than 350 °C, and SAPO-34 displays higher selectivity than ZSM-5 (16% vs 3%), in line with the typical methanol-to-hydrocarbons (MTH) mechanism.^23^ Looking in detail at the PdZn/ZrO_2_+SAPO-34 system, the achieved propane selectivity is higher than the MeOH one of the stand-alone PdZn/ZrO_2_ catalysts at 350 °C (16% vs 7%). Interestingly, MeOH selectivity is overlapped with the equilibrium line, indicating that at these conditions the reaction is limited by the reaction thermodynamics (Figure S2a). Conversely, propane selectivity is substantially higher than MeOH equilibrium, suggesting an equilibrium displacement when the multifunctional system is assembled. Therefore, we can conclude that SAPO-34 seems to be a more promising candidate to produce propane than ZSM-5. Moreover, although there are thermodynamics restrictions at high temperature, it seems that an operation temperature of 350 °C is needed for the MeOH conversion to occur in both SAPO-34 and ZSM-5.

Next, the more promising PdZn/ZrO_2_+SAPO-34 system was studied at 350 °C using different space velocity values (1500, 3000, 6000, and 12000 mL g^–1^ h^–1^) and pressures (30, 40, and 50 bar). The results are summarized in Figure 1b. As expected, CO_2_ conversion increases with decreasing space velocity and increasing pressure. However, CO selectivity also decreases with space velocity, reaching a minimum value of 25% at 50 bar and 1500 mL g^–1^ h^–1^. This behavior is unexpected as, for most of the state-of-the-art CO_2_ to MeOH catalysts, the opposite trend is observed and higher MeOH selectivity is usually obtained at higher space velocity value.^24^ Hence, these data may suggest that CO-involving reactions play a key role using the PdZn/ZrO_2_+SAPO-34 system, because of a limitation in its formation or a consumption of the formed CO to produce more methanol. Moreover, from the data at 50 bar and 1500 mL g^–1^ h^–1^, a total selectivity to propane higher than 50% can be observed, with a CO_2_ conversion close to 40% and only 25% of CO selectivity. To the best of our knowledge, this is the highest total selectivity reported for a single C_2+_ hydrocarbon during CO_2_ hydrogenation at meaningful conversion levels.^11^ Additionally, thanks to the Pd hydrogenating effect and as intended, the paraffins account for more than 99.9% of the products, greatly facilitating the product separation in a potential industrial process. This later can be better observed if we look in detail at the CO free hydrocarbon distribution (Figure 1c). Figure S2 shows conversion/selectivity plots with these experiments being compared with the above-discussed CO_2_-to-methanol equilibrium. The presence of acid sites in SAPO-34, in close proximity to the methanol-forming PdZn/ZrO_2_ catalyst, leads to rapid conversion of methanol into hydrocarbons (mainly propene). Consequently, the methanol concentration remains below the equilibrium limit, and the selectivity to propane (formed from the fast hydrogenation of propene) is significantly above the equilibrium line at 350 °C and 30 (Figure S2a), 40 (Figure S2b), and 50 bar (Figure S2c). Otherwise, the selectivity to methanol was clearly restricted by equilibrium under these conditions (Figure S2a, orange dot).

**Figure 2:**
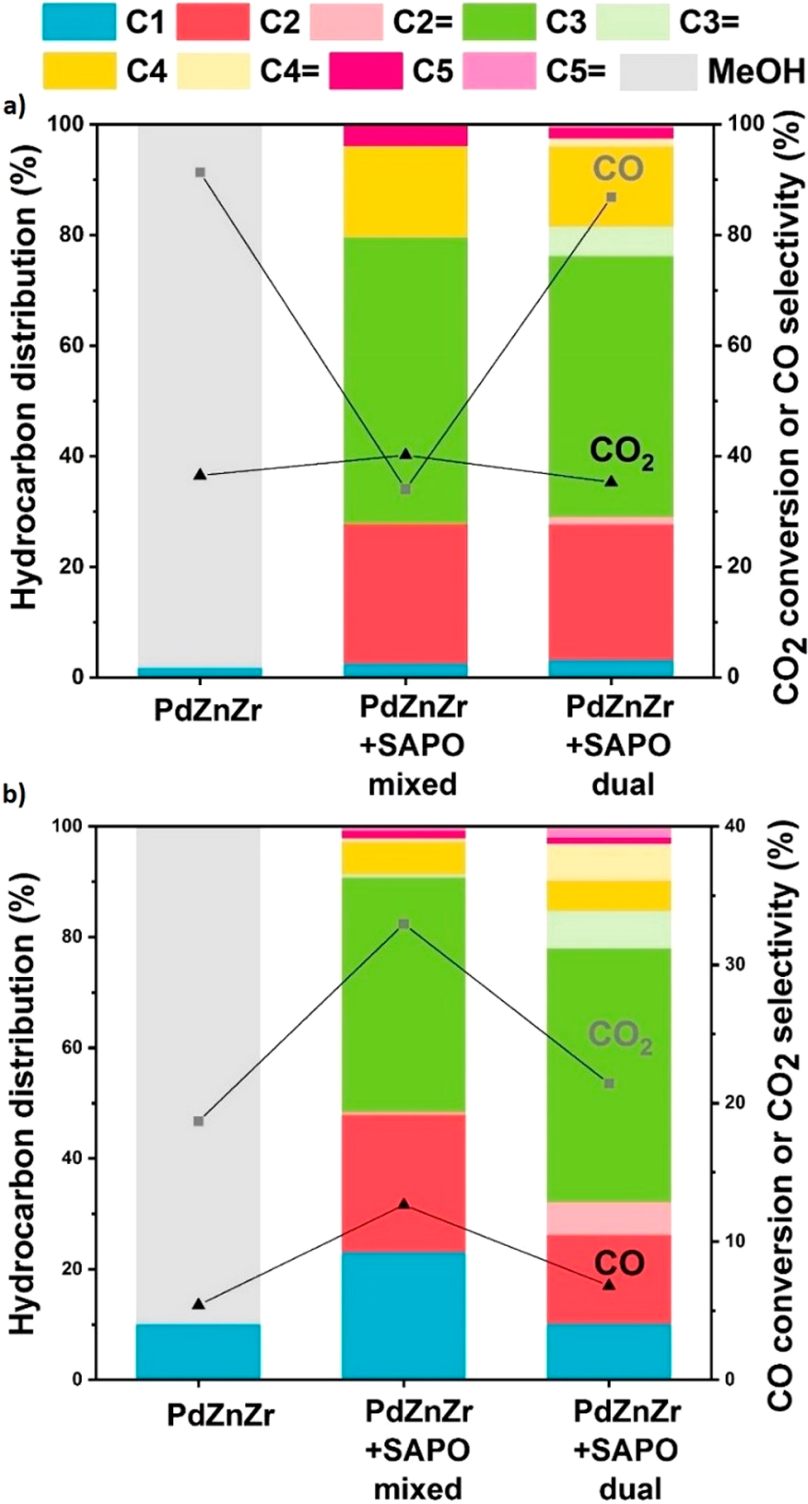
Catalytic performance of the PdZn/ZrO_2_ catalyst and the PdZn/ZrO_2_+SAPO-34 combined system: (a) CO_2_ and (b) CO conversion to hydrocarbons. CO_*x*_:H_2_ 1:3, 350 °C, 30 bar, 3000 mL g^–1^ h^–1^.

To shed light on the strikingly low CO selectivity and the CO role, additional experiments with both CO_2_ and CO feeds were performed comparing the original PdZn/ZrO_2_+SAPO-34 mixed system with the stand-alone PdZn/ZrO_2_ catalyst and the multifunctional system in dual bed configuration. The results are summarized in Figure 2. Considering first the CO_2_ feed (Figure 2a), the rather stable CO_2_ conversion, and the huge decrease in CO selectivity (from 95% to 35%), only observed when mixing PdZn/ZrO_2_ with SAPO-34, a lower CO formation rate in the presence of SAPO-34 is suggested. The similar CO selectivity with similar CO_2_ conversion in the dual bed setup confirms that, indeed, the intimate mixture of both components is needed to displace equilibrium. Here, the CO-forming PdZn/ZrO_2_ will compete with the hydrocarbon-forming SAPO-34 for methanol and, due to the rapid transformation of methanol to hydrocarbons over SAPO-34, the CO_2_/MeOH/CO equilibrium system would be shifted to minimize CO formation in the mixed bed case. The higher olefin-to-paraffin ratios in the dual bed configuration are the result of the higher hydrogenation ability of PdZn/ZrO_2_ compared to SAPO-34 as we initially intended. At the same time, the dual bed data demonstrate that SAPO-34 has substantial hydrogenation activity in the presence of surplus H_2_. This is well in line with prior literature over SAPO-34 in the presence of H_2_.^25^

Considering the CO feed (Figure 2b), CO conversion is substantially lower than that of CO_2_, but with higher methanol selectivity compared to CO_2_ over the PdZn/ZrO_2_ alone. This is in line with process thermodynamics (the lower the conversion, the higher the MeOH selectivity) but also suggests that CO limits the rate of the reaction. Surprisingly, here the CO_2_ selectivity shows a different trend and it is maximized in the mixed bed PdZn/ZrO_2_+SAPO-34 configuration. CO conversion is also increased in this mixed bed configuration. We attribute this result to another equilibrium displacement. This time, the water–gas shift (WGS) is promoted because of the water presence. Converting the MeOH generated on the PdZn/ZrO_2_ to hydrocarbons on the SAPO-34 generates great amounts of water that can favor the WGS on the hydrogenating catalyst, thus increasing the CO conversion and selectivity to CO_2_. Summing up, from these results, we can confirm the clear effect of CO and WGS in the mixed bed configuration when both catalytic functions are intimately mixed together and the equilibrium can be shifted.

### 3.2 Kinetic Modeling of the PdZn/ZrO_2_+SAPO-34 System

**Figure 3:**
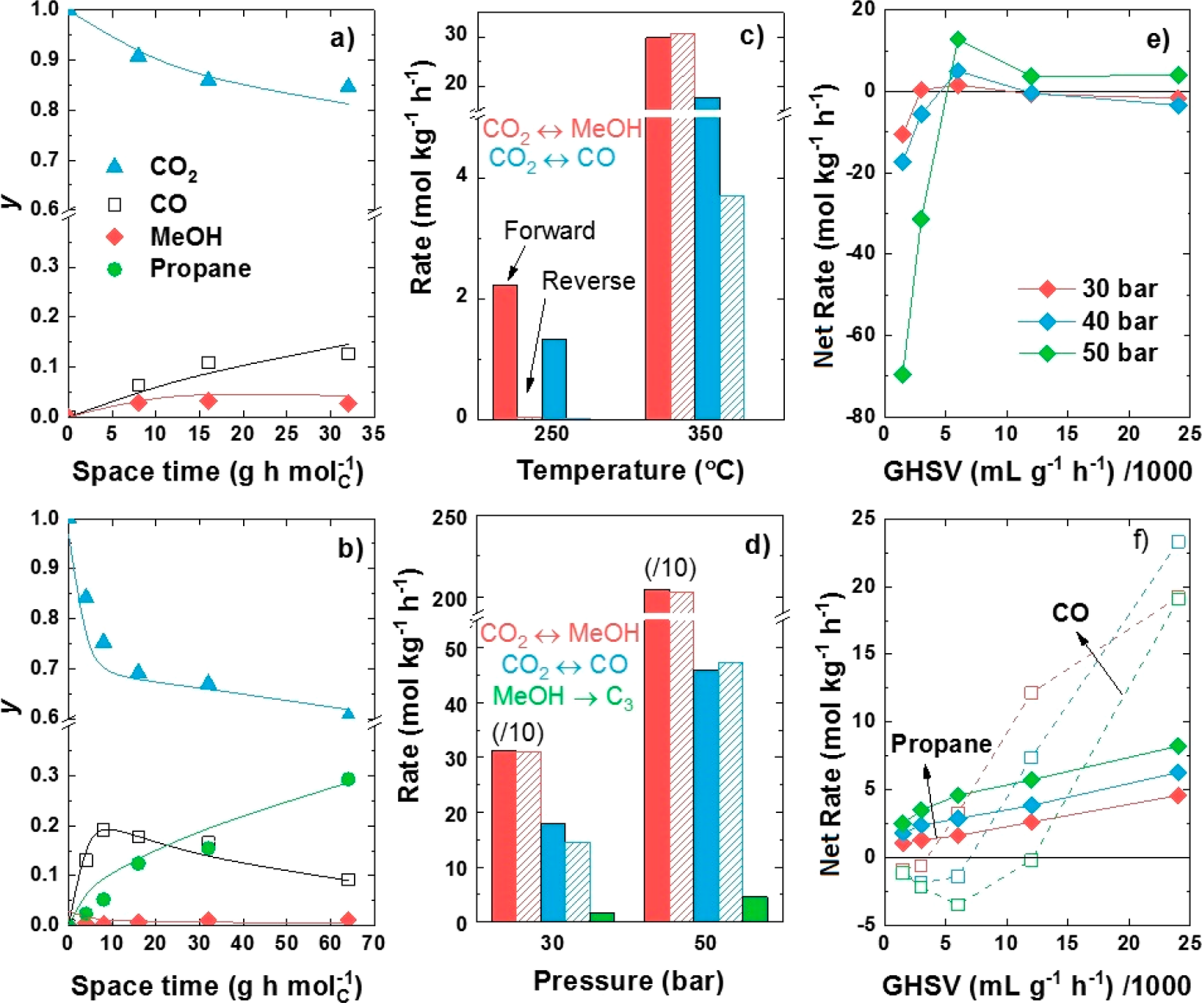
Experimental data fitting of (a) CO_2_ to methanol over the PdZn/ZrO_2_ catalyst at 300 °C and 30 bar and (b) CO_2_ to propane over the PdZn/ZrO_2_+SAPO-34 system at 350 °C and 50 bar. (c) Comparison of reaction rates for methanol and CO formation over the PdZn/ZrO_2_ catalyst at 250 and 350 °C and 30 bar (12000 mL g^–1^ h^–1^), and (d) influence of propane formation on these rates over the PdZn/ZrO_2_+SAPO-34 system at 350 °C and 30 and 50 bar (6000 mL g^–1^ h^–1^). Evolution with GHSV of the net formation rate of (e) methanol and (f) CO and propane over the PdZn/ZrO_2_+SAPO-34 system at 350 °C.

Because of the relevant role of equilibrium and thermodynamics in the overall process and the observed advantages of intimate mixing both functions, a kinetic modeling study was carried out comparing the performances of the stand-alone PdZn/ZrO_2_ catalyst and the PdZn/ZrO_2_+SAPO-34 system. More details on the reaction network and kinetic equations can be found in the Supporting Information. Experimental data fitting at selected conditions can be observed in Figure 3a, b for both catalysts at the optimal conditions for the production of methanol and propane, respectively. Moreover, the fitting of all experimental data can be found in Figure S3. A clearly differentiated trend is observed. CO_2_ is converted to CO and MeOH over the stand-alone catalyst, with the concentration of MeOH decreasing with space time and that of CO reaching a saturating trend at 350 °C (Figure S3c). Otherwise, in line with our previous claims, CO shows a maximum when both functions are mixed, accentuated at 50 bar (Figure 3b). As expected, the concentration of propane increases with space time. As propane is formed only from MeOH, this can only be explained by shifts on CO_2_/CO/MeOH equilibria caused by the presence of SAPO-34. This can be more clearly understood when the estimated reaction rates for the CO_2_-to-MeOH and rWGS reactions are compared (Figure 3c, d). The evolution with space time of the individual reactions rates at all tested temperatures and pressures are shown in Figures S4 and S5.

At low temperature and low CO_2_ conversions (250 °C, Figure 3c), reactions are not limited by equilibrium, as was well-predicted by thermodynamics studies (Figure S4). Forward reactions are remarkably promoted but with low rate values. When temperature is increased, rate values increase but forward/reverse rates for CO_2_/MeOH equilibrium are, in practice, the same, confirming the above-discussed thermodynamics constraint at this high temperature. A comparison of these rates with those calculated for the PdZn/ZrO_2_+SAPO-34 multifunctional system at the same conditions and half GHSV (same CO_2_-to-PdZn/ZrO_2_ ratio, Figure 3d) shows a ten-times increase in MeOH formation (in equilibrium) because of its consumption to form propane, but also a significant increase in the reverse rate of CO formation (ca. 18 mol kg^–1^ h^–1^, the forward reaction, ca. 4 vs ca. 14 mol kg^–1^ h^–1^, the reverse reaction). The better performance at 50 bar is explained by the minimum formation of CO due to the CO_2_/CO reaction being also in equilibrium, which is caused by the promotion of the propane formation rate. This result suggests a substantial modification of the CO_2_/CO/MeOH equilibrium but also, as expected, a limitation in propane production due to the system thermodynamics.

The selective methanol conversion can be better observed in the evolution with GHSV of MeOH (Figure 3e), CO, and propane (Figure 3f) net formation rates. Those corresponding to the stand-alone PdZn/ZrO_2_ catalyst can be found in Figure S6. At high GHSV values, MeOH formation rate tends to zero, with maximum rates for CO and propane formation. This coincides with the lowest CO_2_ conversion values shown in Figure 1b. At conditions of optimized CO_2_ conversion with low GHSV values, the net rate of MeOH decreases to negative values, also leading to a significant decrease in CO net formation rate. As a consequence, the propane rate is selectively higher, explaining the highest selectivity to this product at these conditions (Figures 1b and 3f). Moreover, all these trends are maximized when the pressure is increased, with 50 bar being the optimal pressure to enhance propane selective formation.

As previously discussed, the conversion of MeOH into propane takes place through the dual-cycle mechanism and a fast hydrogenation of the formed olefins. This industrial process over SAPO-34 suffers a well-known fast deactivation due to the formation of coke within the zeotype cages. For this reason, deactivation can also play a role in the view of the industrial implementation of the tandem process presented herein. Therefore, the effect of in situ regeneration at 600 °C was studied with a 5% O_2_ in N_2_ stream for 5 h (Figure S7). The catalytic system is rather stable for at least 48 h and only at very high space velocity values (24000 mL g^–1^ h^–1^), deactivation is significant. In particular, after deactivation at 24000 mL g^–1^ h^–1^, the products selectivity is mainly a mix of MeOH and DME with CO (73% MeOH, 26% DME and 1% CH_4_), whereas the CO_2_ conversion remains invariable, further corroborating that the SAPO-34 component is the one being deactivated. However, the in situ regeneration worked for all samples and the initial activity was regained after the regeneration cycle at 600 °C.

### 3.3 Characterization of the Multifunctional Catalysts

The powder X-ray diffraction (PXRD) pattern of the as prepared PdZn/ZrO_2_ sample is shown in Figure S8. The sample shows a diffraction pattern of zincite (ZnO), PdO, and tetragonal/cubic ZrO_2_. Concerning the ZrO_2_ structure, even if cubic and tetragonal polymorphs are not discernible because of crystallite-induced peak broadening, their distinction is out of the scope of the present work. Indeed, as shown hereafter, ZrO_2_’s major role is to be an active support of PdZn alloy for CO_2_ adsorption through carbonates formation, making the identification of a single phase/mixture of monoclinic/tetragonal (cubic) polymorphs the most relevant detail.^26^ Energy-dispersive X-ray spectroscopy (EDS) shows a molar composition of 2% Pd, 13% Zn, and 85% Zr, close to the theoretical synthesis value (Table S2).

Nitrogen adsorption-desorption isotherms of both SAPO-34 and ZSM-5 zeolites are depicted in Figure S9. The detailed textural properties are summarized in Table S3. SAPO-34 displays a microporous type I isotherm and ZSM-5 a micro-mesoporous type IV isotherm. The BET surface of SAPO-34 is estimated to be 770 m^2^/g with 762 m^2^/g of micropores, whereas for ZSM-5, it is estimated to be 417 m^2^/g with 296 m^2^/g of micropores. NH_3_-TPD profiles of both zeolites are depicted in Figure S10. SAPO-34 displays only one peak at ca. 400 °C, whereas ZSM-5 displays the two characteristic peaks of weak (Lewis) and strong (Brønsted) acid sites at 225 and 425 °C, respectively.^27^

#### 3.3.1 Local Structure and Electronic Properties of Pd and Zn Species

**Figure 4:**
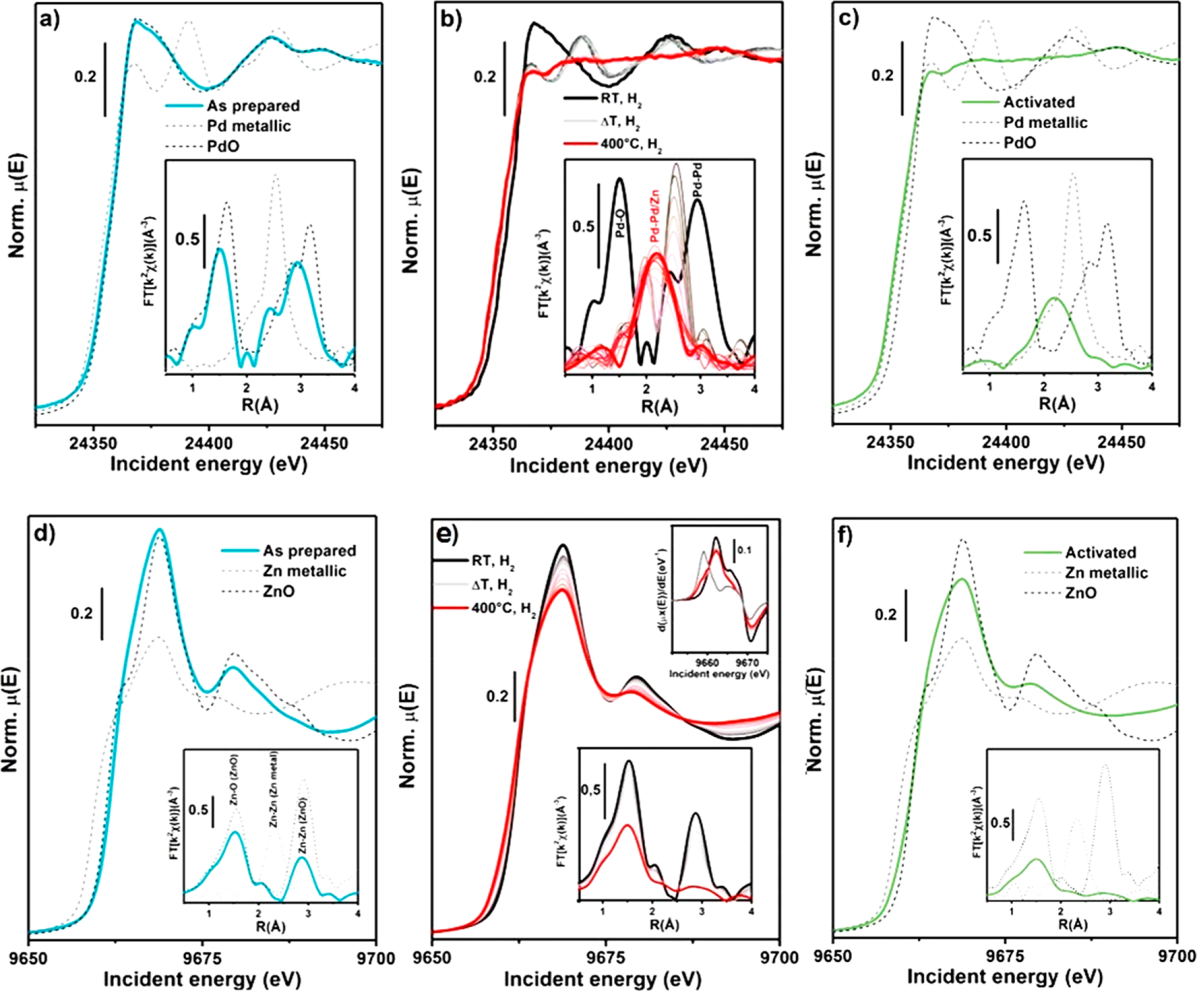
Pd K-edge, Zn K-edge XANES (main panels), and EXAFS (insets) spectra of PdZn/ZrO_2_+ZSM-5 catalyst. XAS spectra for relevant reference compounds are also reported as dashed lines. (a) Pd K-edge for as prepared catalyst. (b) Pd K-edge for catalyst during activation (RT-400 °C) under H_2_ gas flow. (c) Pd K-edge for catalyst after activation at 400 °C under H_2_ atmosphere. (d) Zn K-edge for as prepared catalyst. (e) Zn K-edge for catalyst during activation (RT-400 °C) under a H_2_ gas flow. Top right inset: first derivative of the XANES spectra for as prepared catalyst (black), activated catalyst (red), reference Zn(0) metal foil (light gray). (f) Zn K-edge for catalyst after activation at 400 °C under a H_2_ gas flow. For clarity of comparison, the Pd metallic foil EXAFS signal was rescaled by a factor of 1/2. The EXAFS spectra reported in the bottom insets have been obtained by transforming the corresponding *k*^2^χ(*k*) EXAFS function in the 2.5–11.0 Å^–1^ range.

X-ray absorption spectroscopy (XAS) was initially performed on the PdZn/ZrO_2_ catalyst physically mixed with ZSM-5 aiming to characterize, in an element-selective way, Pd- and Zn-containing species formed in a model multifunctional system, in its as-prepared state and upon activation. The XAS spectra reported in Figure 4 show how electronic and structural features of both Pd and Zn change considerably when the catalyst is subjected to the activation treatment. Indeed, both XANES (main panels) and EXAFS spectra (insets) show substantial modifications at high temperature in the presence of H_2_.

Considering the Pd K edge, the as-prepared catalyst (Figure 4a) presents the typical XANES (edge position, postedge resonances) and EXAFS (Pd–O bond in first coordination shell) features of the reference PdO, consistently with PXRD results (section 3.1). During activation (Figure 4b, from ca. 50 °C up to ca. 200 °C) Pd(II)-to-Pd(0) reduction is underlined by (i) shift of the absorption edge position to lower energy, (ii) rapid change of the oscillation in the XANES from PdO-like to those resembling metallic Pd (Figure 4a, b light gray line), and (iii) intensity loss of the Pd–O first coordination shell in the phase-uncorrected EXAFS (inset Figure 4b) and shift to lower *R* values of the Pd–Pd second coordination shell. As the temperature rises (300 °C), the oscillations in the XANES get flatter and EXAFS evolves into a single broad peak around 2.15 Å in the phase-uncorrected spectrum (Figure 4b red curve). These features were already observed and well reported in the case of other PdZn systems and ascribed to the formation of β_1_-PdZn alloy.^21,28−35^ The EXAFS fit on the activated catalyst spectrum, reported in Figure S11, further confirmed the alloy formation. The fit obtained considering the simultaneous presence of Pd–Zn and Pd–Pd scattering paths well reproduced the experimental data. The magnitude and the imaginary parts of the FT-EXAFS spectra (Figure S11a,b) presented a broad peak and a single oscillation, respectively. Analysis of the imaginary parts of the Pd–Pd and Pd–Zn scattering paths optimized in the fit (Figure S11c) highlighted a wide overlap of the two contributions, justifying the observed broadening in the experimental spectra (Figure S11a, b). Despite their overlap, Pd–Zn and Pd–Pd paths have a stronger contribution at lower and higher radial distances, respectively, leading to evaluation of a shorter Pd–Zn and a longer Pd–Pd path, the values of which are reported in Table S4 and confirmed the PdZn alloy formation.^21,28^ σ_2_ values are consistent with the thermal contribution at 400 °C and are comparable with literature results obtained at similar temperatures.^21,28^

Moving to the Zn K-edge, the as-prepared catalyst (Figure 4d) essentially presents the same XANES and EXAFS features of the reference ZnO, also in line with PXRD analysis of the as-prepared PdZn/ZrO_2_ phase. A careful observation of the EXAFS data unveils a lower intensity of the second shell peak in the catalyst with respect to the reference oxide (inset Figure 4d), indicating a higher concentration of defects in the former. From the Zn perspective, the activation protocol causes (i) a pronounced decrease of the signal intensity in both the XANES and EXAFS region and (ii) a subtle red-shift of the edge energy position, better observed from the growth of a shoulder at low energy values in the XANES first derivative, matching the first maximum for Zn(0) metal foil (top right inset Figure 4e). Zn species are present in the activated sample (Figure 4f), and therefore they dominantly occur as a highly defective ZnO phase with a minor Zn(0) contribution ascribable to the fraction of Zn taking part to the formation of PdZn alloy. Despite its nature as a bulk-sensitive technique, in the case of ion-exchanged zeolite/zeotype systems, XAS becomes extremely sensitive to the absorber atom local environment.^36−39^ In the case of other PdZn/zeolite combined systems, Zn was observed to diffuse within the zeolite,^29^ modifying the Zn local environment and leading to characteristic Zn K-edge spectra well reported in the literature.^38^ In the case of the system here investigated, i.e., PdZn/ZrO_2_+zeolite, fingerprints of Zn-exchanged zeolite were not observed,^38^ suggesting that impregnating Pd and Zn over ZrO_2_ stabilizes the former atoms, avoiding their further diffusion into the zeolitic component.

#### 3.3.2 Surface Interactions with Key Reactants and Molecular Probes

Differently from XAS, which provided an element-selective view on Pd- and Zn-containing species in a representative combined system, FT-IR analysis was focused on the stand-alone PdZn/ZrO_2_ system. Exploring its interaction with key reactants/molecular probes (H_2_, CO_2_, and CO), we aimed at confirming and further deepening two major issues related to the CO_2_-hydrogenation functionality, namely activation-driven oxygen vacancies formation and nature of the PdZn phase.

Zinc oxide phase plays a key role for both intermediate stabilization and H_2_ heterolytic splitting thanks to its propensity to form stoichiometric defects (such as oxygen vacancies).^40−42^ In our case, the presence of a ZnO phase is shown by XAS and XRD results. Specifically, according to XAS, after the activation a highly defective ZnO phase is formed, together with the PdZn alloy. Hence, CO_2_ hydrogenation can be due to the oxygen-vacancy formation in the defective ZnO phase. To investigate the formation of oxygen vacancies, different IR measurements were performed in H_2_ at different temperatures, as well as under oxidizing conditions for comparison purposes.

To understand the IR results, it is important to underline that lattice defects, such as oxygen vacancies (*V*_O_), make ZnO a semiconducting material.^43−45^ Neutral *V*_O_ shows two trapped electrons located in levels at 0.05 and 0.18 eV below the conduction band (C.B.). The first electronic level is very near to the bottom of the C.B., so that the majority of electrons can be moved to the C.B. at room temperature to produce monoionized oxygen vacancies (*V*_O_^+^). The second ionization of *V*_O_ can be promoted by IR, and thus it is possible to observe the photoionization of monoionized oxygen vacancies. Typically, pure ZnO shows a broad absorption band centered at about 1450 cm^–1^, i.e. 0.18 eV, after reduction treatments.^45^ Interaction with H_2_ can create *V*_O_^+^ following two main pathways: (i) the filling with an electron of pre-existing bi-ionized *V*_O_ (*V*_O_^2+^) by consuming adsorbed oxygen species, such as O_2_^–^, O^–^, O_2_^2–^; (ii) the generation of new *V*_O_^+^ extracting lattice oxygen ions from the surface, but this route occurs only at high temperature. The IR method is not able to discriminate the two routes for *V*_O_^+^ formation.

Figure S12 displays different spectra obtained in H_2_ at different temperature from 50 to 400 °C. The broad absorption band related to monoionized oxygen vacancies is well evident. The band increases in intensity up to 150 °C, losing intensity at higher temperature. The intensity loss can be ascribed to the PdZn alloy formation evidenced by XAS measurements (reduction of Zn^2+^ to Zn^0^ occurs at the expense of electrons trapped in V_O_^+^). As a matter of fact, the formation of metallic Zn at this temperature is not expected, but it could be favored by Pd presence and alloy formation. The band is centered at about 1250 cm^–1^, which corresponds to monoionized oxygen vacancies at about 0.15 eV under the C.B., very near to the ionization energy observed for pure ZnO. The obtained results, in agreement with XAS findings, demonstrate the presence of a highly defective ZnO-like phase.

**Figure 5:**
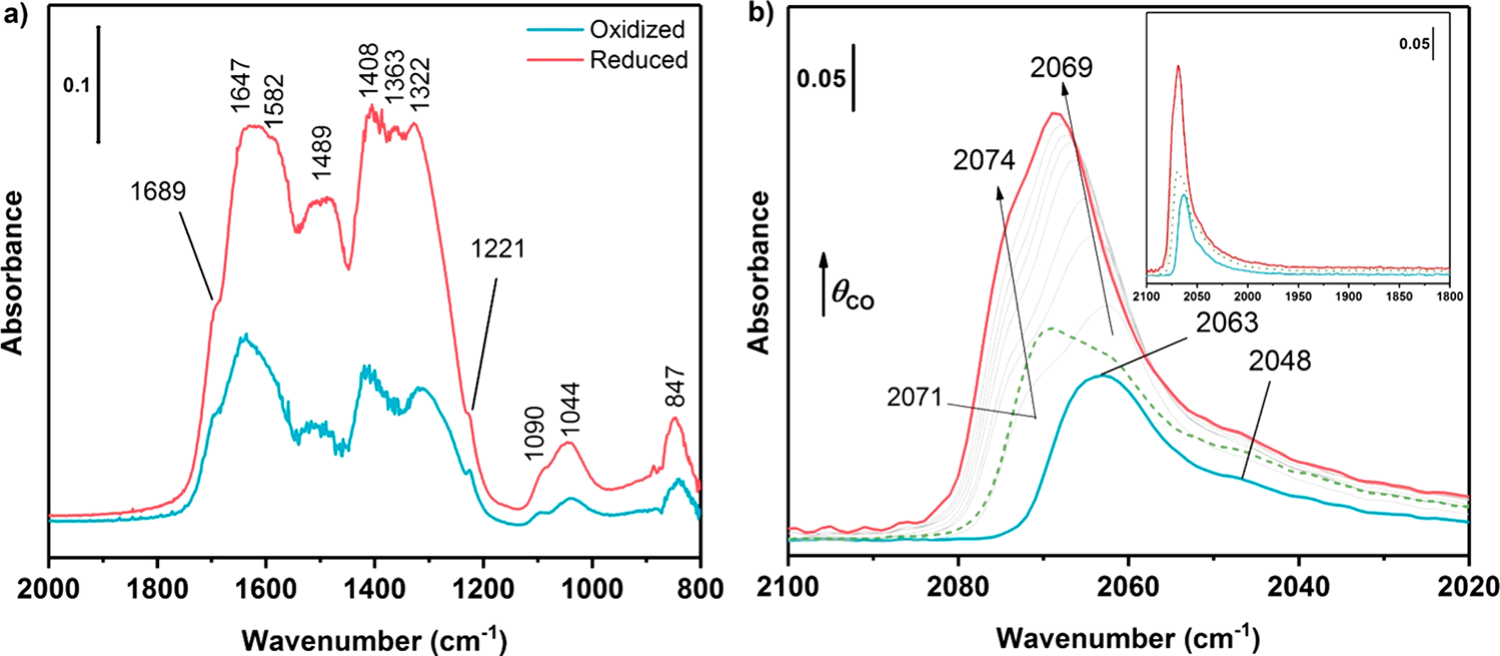
(a) FT-IR spectra of CO_2_ adsorbed at RT on oxidized and activated PdZn/ZrO_2_ at equilibrium pressure of 20 mbar. (b) FT-IR spectra of CO adsorbed at RT on activated PdZn/ZrO_2_. Spectra were acquired at increasing dosage of CO up to 20 mbar (from blue to red line) and after outgassing (green dashed line).

To explore the interaction/adsorption of CO_2_ with the catalyst surface, we followed adsorption at room temperature on both oxidized and H_2_-activated PdZn/ZrO_2_ by FT-IR spectroscopy and spectra, reported in Figure 5a. All the shown bands can be assigned to different carbonate species. These species could be formed on both the ZnO and ZrO_2_ phase; however, CO_2_ adsorption on ZnO^46,47^ and tetragonal ZrO_2_^48^ gives carbonates with spectral features different from those shown in Figure 5a. In particular, tetragonal zirconia gives an appreciable amount of polydentate bridging carbonates that are not present in our case. Bare ZnO gives an appreciable amount of bicarbonates that are present in very small amounts in our case. None of them forms the bridged and monodentate species that are present for the PdZn/ZrO_2_ catalyst. More specifically, the following adsorbed species can be identified:^47−53^ (i) bicarbonates, weak bands at 1689 and 1221 cm^–1^, assigned to ν(C=O) and δ(C–O–H) modes, respectively; (ii) bridged carbonates, bands at 1647, 1322, and 1044 cm^–1^, assigned to ν(C=O), ν_asym_(O–C–O), and ν_sym_(O–C–O) modes, respectively; (iii) bidentate carbonates, bands at 1582, 1363, and 1044 cm^–1^, assigned to ν(C=O), ν_asym_(O–C–O), and ν_sym_(O–C–O) modes, respectively; (iv) monodentate carbonates, bands at 1489, 1408, and 1090 cm^–1^, assigned to ν_asym_(O–C–O), ν_sym_(O–C–O), and ν(C–O), respectively. The band at 847 cm^–1^ can be related to the δ(O–C–O) mode of both monodentate and bidentate carbonates, as mentioned by some works on different oxides.^54,55^

Spectral features obtained for the oxidized sample are the same obtained for the activated one, but the overall intensity of the bands in Figure 5a shows that there are much more carbonates on the activated sample when compared with the oxidized one. The reason could reside in the stoichiometric defect formation: reduction generates oxygen vacancies on the surface of ZnO, giving a highly defective phase more suitable for carbonate formation. In light of this result, we reasonably ascribe the formation of carbonate species mainly to CO_2_ adsorption on this phase. Different spectral features with respect to bare ZnO carbonates^47,56^ can be related to the fact that ZnO is a supported phase in our system. This causes the presence of huge amount of surface defects, such as edges, corners and kinks, beside stoichiometric defectiveness, which is characteristic of ZnO itself. Surface defects are able to create high heterogeneity of surface-adsorbed species. Moreover, the interaction with both ZrO_2_ and Pd reasonably contributes to carbonate species heterogeneity.

CO adsorption at room temperature was also performed to characterize metallic Pd and explore its interaction to CO. The adsorption was carried out on activated PdZn/ZrO_2_, i.e., after reduction in H_2_ at 400 °C. Figure 5b reports several spectra obtained at increasing CO coverage (θ_CO_) corresponding to increasing CO pressure up to 20 mbar: an asymmetric band in the region between 2080 and 2020 cm^–1^ appears and increases in intensity upon increasing pressure. The band lies in the region of Pd^0^ linear carbonyls^48,57^ and more specifically, it appears constituted by different components: the main peak at 2063 cm^–1^ that shifts to 2069 cm^–1^ at the maximum CO coverage and two shoulders at 2071 cm^–1^ (2074 cm^–1^ for the maximum θ_CO_) and 2048 cm^–1^. As a matter of fact, the frequencies reached at the maximum coverage are quite lower with respect to the characteristic ones of Pd^0^ linear carbonyls. According to literature,^58−60^ the lower frequencies can be ascribed to CO on Pd sites in the PdZn alloy. Indeed, the dilution of Pd in the alloy deletes the dipolar coupling and changes in the electronic properties of Pd, induced by the alloying, can slightly increase π-backdonation.

To assign the different components, it is worth remembering that the Pd^0^–CO bond shows strong π-backdonation character; as a consequence, the lower the band frequency the more coordinatively unsaturated the site.^61^ Hence, the shoulder at 2048 cm^–1^ is assigned to CO adsorbed on highly defective Pd^0^ sites, such as corners, the main band at 2063–2069 cm^–1^ to less defective sites, such as edges, whereas the shoulder at the higher frequency can be related to terrace sites.

As for the blue shift observed by increasing coverage, it can be explained by two phenomena: the dipolar coupling and the “chemical effect”. The first one is predominant for terrace sites on regular facets but, in our case, it can be neglected due to the presence of Pd dilution by alloying with Zn, as evidenced by XAS measurements. The second one is caused by the decrease in π-backdonation as the number of adsorbed CO molecules increases, so that the higher the θ_CO_, the lower the donated electron density per each adsorbed molecule. Therefore, upon increasing coverage, the π-backdonation contribution to all adsorbed CO molecules becomes weaker and weaker, and then an increase in ν(C≡O) is observed.

After outgassing (dashed-green line in Figure 5b), the intensity of the peak is drastically reduced but not totally brought down, showing a certain stability of Pd^0^–CO at room temperature. It is important to note that the outgassing cancels out all of the effect due to the θ_CO_. Hence, the peaks are shifted back to their original frequency, with slightly different relative intensities of the three main absorptions. Pd is also known to form a considerable amount of bridged carbonyls on different supports when reduced to metallic Pd.^48,62^ In our case, it is worth noting the absence of bridged Pd–carbonyls in the region between 2000 and 1800 cm^–1^ (inset Figure 5b). This highlights the absence of neighboring Pd atoms, confirming the PdZn alloy formation, in agreement with XAS results.

#### 3.3.3 Morphological/Chemical Insights on Used Multifunctional Catalysts

**Figure 6:**
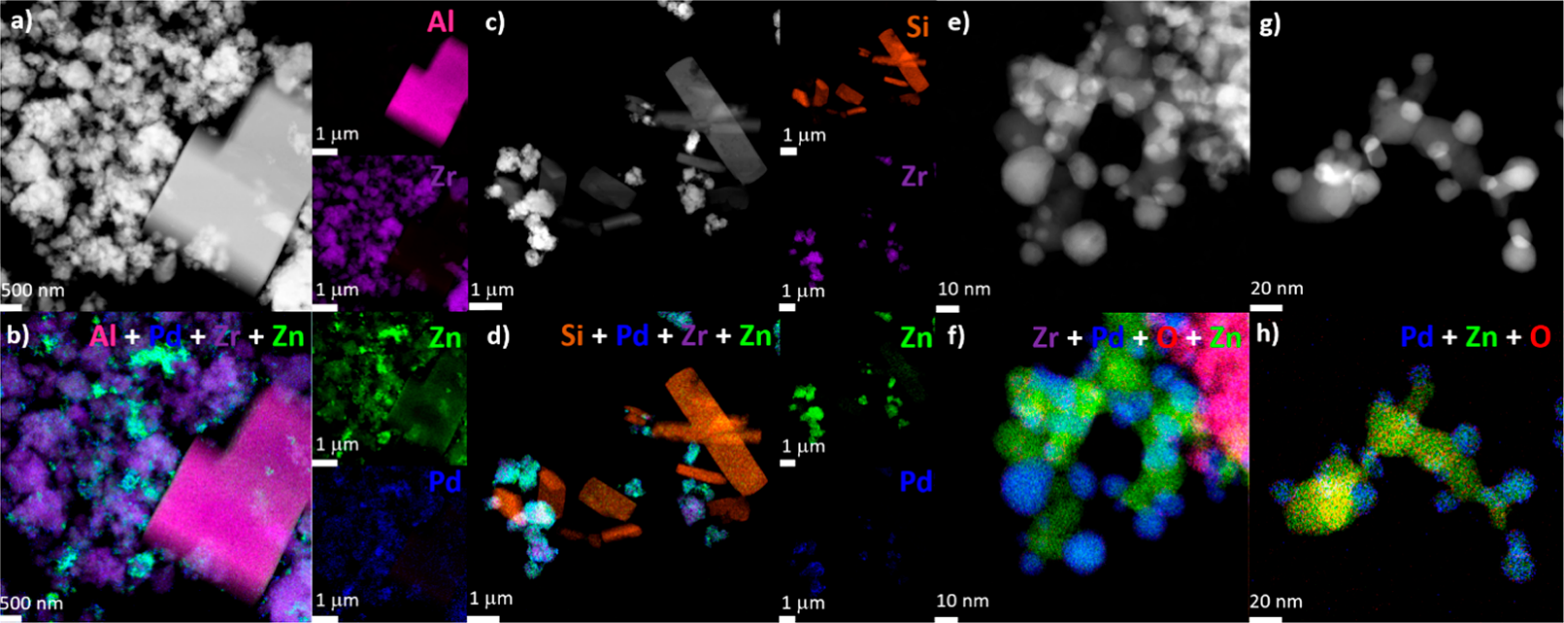
Low-magnification HAADF-STEM imaging of PdZn/ZrO_2_ catalysts mixed either with (a) SAPO-34 and with (c) ZSM-5 crystals and (b, d) related elemental maps built with Kα emission lines provided by Al, Si, Pd, Zr, and Zn atoms. Magnification of the PdZn/ZnO nanoparticles observed at the edge of the ZrO_2_ support when mixed either with (e) SAPO-34 and with (g) ZSM-5 crystals. (f, h) Associated overlaps of elemental maps.

Imaging by high-angle annular dark-field scanning transmission electron microscopy (HAADF-STEM) was performed to investigate the morphological properties of the PdZn/ZrO_2_ catalyst mixed either with SAPO-34 or with ZSM-5, recovered after catalytic testing for 24 h at 350 °C, 30 bar, and 12000 mL g^–1^ h^–1^. The low-magnification micrograph presented in Figure 6a shows a typical juxtaposition of large cubic SAPO-34 crystals with the PdZn/ZrO_2_ function showing as nanoparticle agglomerates. Furthermore, Zr, Zn, and Pd elemental maps computed from X-ray fluorescence spectroscopy (STEM-EDX) reveal that most of the Pd and Zn atoms are distributed at the periphery of the ZrO_2_ support (Figure 6b). Similar observations are also recorded when the PdZn/ZrO_2_ catalyst is mixed with ZSM-5 (Figure 6c, d). Besides, there is no clear signal from the X-ray fluorescence spectra that would suggest a quantitative migration of Pd and Zr atoms over or into SAPO-34 and ZSM-5 crystals during CO_2_ hydrogenation. However, a small X-ray emission from Zn atoms was detected in both case (<1 wt %). The nature of those Zn species was difficult to pinpoint given their low abundance and the thickness of the SAPO-34 and ZSM-5 crystals.

Magnification on the edge of the ZrO_2_ support shows nanoparticles in 5–30 nm range (Figure 6e, g). Interestingly, some nanoparticles appear brighter, suggesting the presence of heavier atoms, e.g., Pd. The atomic composition shown on Figure 6f, h) reveals a mixture of ZnO and alloyed PdZn nanoparticles, as suggested by former HAADF imaging and consistently with the spectroscopy results presented in section 3.2. Quantification of the Pd and Zn atomic content at the core of the PdZn alloy by X-ray fluorescence provides a Pd/Zn molar ratio of 1.16 ± 0.15 and 1.27 ± 0.14 for SAPO-34 and ZSM-5, respectively (measurement performed on ca. 20 particles). All the observations described below are identical for both catalysts, mixed either with ZSM-5 or with SAPO-34.

**Figure 7:**
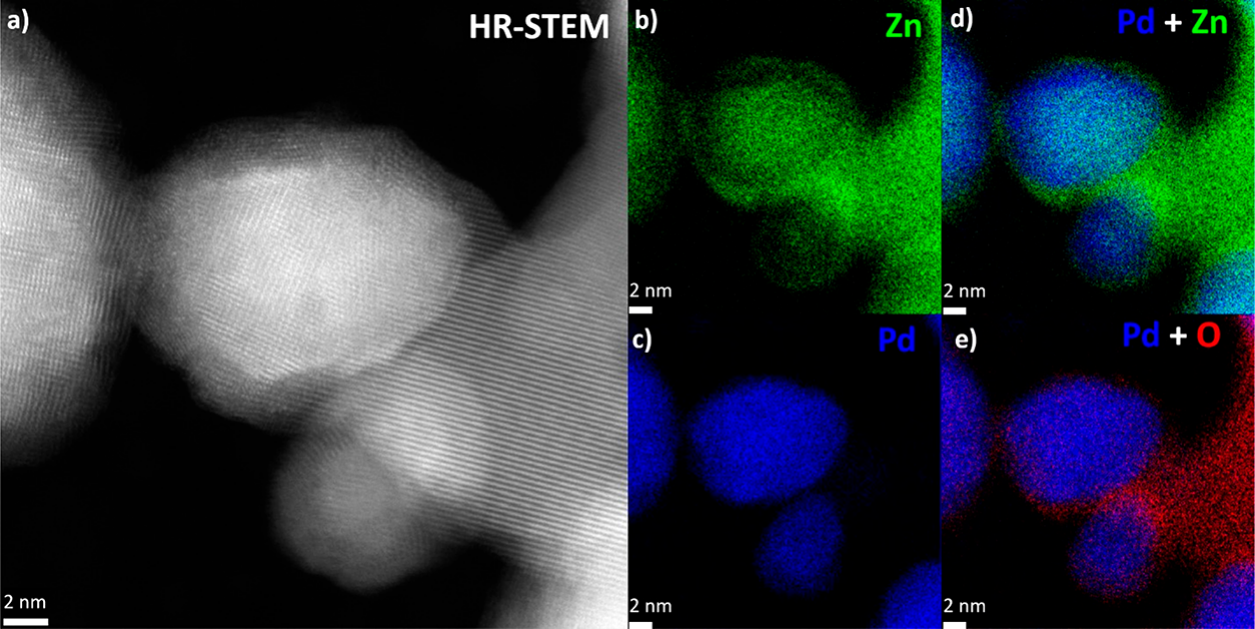
(a) High-resolution HAADF-STEM imaging of a PdZn alloy nanoparticle after CO_2_ hydrogenation. Elemental maps of the same area built with Kα emission lines: (b) Zn, (c) Pd, (d) overlap of Pd and Zn maps, and (e) overlap of Pd and O maps.

High-resolution STEM images highlight a core–shell structure displaying clearly resolved lattice fringes (Figure 7a). The shell is polycrystalline as seen from the various orientation of lattice fringes and has a thickness of about 2 nm. In contrast, the core appears monocrystalline and composed of Pd and Zn atoms. Subsequent elemental mapping provides more details related to the shell composition, which is divided in two parts: (i) a main volume attached to the core where Zn is depleted and metallic Pd majorly remains (Figure 7b–d), (ii) an outer layer composed of zinc oxide (Figure 7b, d, and e). Those compositional characteristics were already reported by Armbrüster et al.^63−65^ for PdZn/ZnO catalysts used with methanol steam reforming (i.e., the inverse reaction: CH_3_OH + H_2_O → 3 H_2_ + CO_2_). They have shown that the oxidation of the PdZn alloy by the CO_2_ gas causes the Zn atoms to leave the intermetallic phase and rise to the surface, forming ZnO patches.

Since a reoxidation of the metallic particles with O_2_ from the air prior to TEM imaging cannot be univocally ruled out,^66^ those results were completed with a imaging study handled without air exposure both after H_2_ activation and CO_2_ hydrogenation. Initially, the sample in its as-synthesized state displays grapes of PdO nanoparticles with a size in the 1–3 nm which are distributed between the ZnO nanoparticles (Figure S14). During H_2_ activation, the PdO nanoparticles within the grapes merges and reduces into 10 to 30 nm metallic nanoparticles (Figure S15). The elemental composition as revealed by STEM-EDS shows clearly the formation of a Pd–Zn alloy at that stage. CO_2_ hydrogenation is also confirmed to induce the formation of the ZnO shell on the surface of the Pd–Zn alloy (Figures S16 and S17). The latter phenomenon was further established by multiple observations in several area of the sample (Figure S18).

To obtain deeper insight into deactivation pathways in bifunctional systems, UV-Raman spectroscopy was also used to characterize the multifunctional system. Figure S13 reports Raman spectra collected at RT on SAPO-34 alone and on the fresh and spent PdZn/ZrO_2_+SAPO-34 combined catalyst. Spectra of the bare SAPO-34 and of fresh PdZn/ZrO_2_+SAPO-34 are almost superimposable. The most intense signals are observed for the spent catalyst, i.e., two intense peaks at 1630 and 1384 cm^–1^. The latter also presents an evident shoulder at higher Raman shifts, roughly centered at 1420 cm^–1^. All these features relate to species trapped in the zeotype cages, identifiable as fingerprints of alkenes (1630 and 1420 cm^–1^ modes)^67−69^ and polycyclic aromatic hydrocarbons (1383 cm^–1^ peak, typical for naphthalene).^70^ TGA analysis was further employed on the spent hybrid system to quantify the amount of coke, showing a 2.96 wt % weight loss in the 300–600 °C region that can be attributed to coke (see Figure 7b). This is evidence for an incipient catalyst deactivation by coking due to the condensation of methanol-to-hydrocarbon reaction intermediates and can justify the decrease in propane production (over SAPO-34) while constant CO_2_ conversion (over the PdZn/ZrO_2_ function) is observed at high GHSV values in Figure S7a.

### 3.4 Reaction Mechanism for the CO_2_ Conversion to Propane

**Figure 8:**
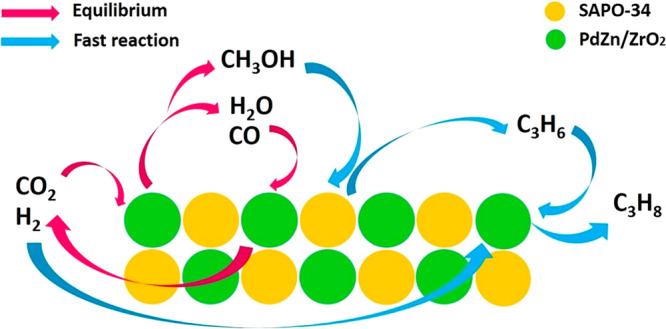
Reaction mechanism for the CO_2_ conversion to propane over the PdZn/ZrO_2_+SAPO-34 system.

On the basis of the above results, we propose a reaction mechanism for the CO_2_ conversion to propane on the PdZn/ZrO_2_+SAPO-34 multifunctional systems (see Figure 8). First, a PdZn alloy is formed during catalyst activation, in intimate contact with oxygen-vacancy rich ZnO particles over the ZrO_2_ support, as demonstrated by XAS and FT-IR (see section 3.3). This alloy, once exposed to the CO_2_-containing reaction feed, develops into a core–shell structure (see Figure 7) with a 2 nm polycrystalline ZnO shell and it is directly responsible of the MeOH formation in the PdZn/ZrO_2_ component.^71−75^ This MeOH formed in the alloy instantly reacts over the SAPO-34 following a classical MTO mechanism with propene as the main product.^76,77^ The instant consumption of MeOH can be ascribed to the intimate mixture of both PdZn/ZrO_2_ and SAPO-34 and ultimately causes a CO_2_/CO/MeOH equilibria displacement, increasing the CO_2_ conversion and reducing drastically the CO selectivity (from 95% to 35%, see Figure 2a). The rapid transformation of MeOH to hydrocarbons (mainly propene) over SAPO-34 tends to minimize the formation rate of CO as a side product over the PdZn/ZrO_2_ catalyst, which can be explained by a shift in the reverse WGSR (Figure 3). Nevertheless, an avoided formation of CO via MeOH degradation in the PdZn/ZrO_2_ due to its transformation to hydrocarbons cannot be discarded. This was suggested by the clear differences in the net formation rates of CO when SAPO-34 was added to the catalytic bed (Figure 3c, d). Moreover, the H_2_O formed as byproduct over SAPO-34 can also displace the reverse WGS equilibrium (reaction 2 in Supporting Information, CO_2_ + H_2_ → CO + H_2_O),^78^ further reducing the CO selectivity. Lastly, the again intimately mixed PdZn/ZrO_2_ rapidly hydrogenates the propene produced over SAPO-34, forming the resulting propane.

## Conclusions

In conclusion, we have developed a highly active and selective PdZn/ZrO_2_+SAPO-34 multifunctional catalyst for the direct conversion of CO_2_ to propane that displays a total selectivity to propane higher than 50% (with 20% CO, 6% C_1_, 13% C_2_, 10% C_4_, and 1% C_5_) and a CO_2_ conversion close to 40% at 350 °C, 50 bar, and 1500 mL g^–1^ h^–1^. These results can be rationalized as a consequence of the combined effects of each component of the multifunctional system. First, the alloy formed during catalyst activation is responsible for the formation of MeOH in the PdZn/ZrO_2_ component. XAS and FT-IR, together with high-resolution HAADF-STEM imaging, demonstrate that this alloy is directly responsible for the initial high CO_2_ conversion and evolves into a core–shell structure with a 2 nm polycrystalline ZnO shell. The MeOH formed in the PdZn/ZrO_2_ component instantly reacts over the SAPO-34 forming propene as main product following a classic MTO mechanism. This rapid MeOH consumption triggers several reaction equilibrium shifts, ultimately boosting the initial CO_2_ conversion and minimizing the CO selectivity. Lastly, the Pd component of the system hydrogenates all the propene formed over the intimately mixed SAPO-34, resulting in a paraffins hydrocarbon selectivity over 99.9%. Our results confirm the great importance of synergies in catalyst development for the production of individual hydrocarbons from CO_2_ with the ultimate goal of facilitating the transition to renewable, non-fossil-fuel-based energy sources.

## Supporting Information Available

CO_2_ hydrogenation thermodynamics (Figures S1 and S2), kinetic model for CO_2_ hydrogenation (Methodology, Table S1 and Figures S3–S6), regeneration of the PdZn/ZrO_2_+SAPO-34 multifunctional catalyst (Figures S7), chemical and textural characterization of catalysts (Tables S2 and S3 and Figures S8–S10), spectroscopic characterization of the PdZn/ZrO_2_ (Table S4 and Figures S11–S13), imaging of the PdZn/ZrO_2_ and PdZn/ZrO_2_+SAPO-34 catalysts (Figures S14–S18) (PDF)
