# Supplementary material for: Multifunctional Catalyst Combination for the Direct Conversion of CO2 to Propane
Source: JACS Au. 2021 Sep 2;1(10):1719–32. doi: 10.1021/jacsau.1c00302 (PMC8549042; doi:10.1021/jacsau.1c00302)
Supplement: Supplementary file 1 — au1c00302_si_001.pdf [file au1c00302_si_001.pdf]

SUPPORTING INFORMATION

A multifunctional catalyst combination for the direct conversion of CO_2 to propane

Adrian Ramirez[1], Pierfrancesco Ticali[2], Davide Salusso[2], Tomas Cordero-Lanzac[3], Samy Ould-Chikh[1], Christian Ahoba-Sam[3], Aram L. Bugaev[4], Elisa Borfecchia[2], Sara Morandi[2], Matteo Signorile[2], Silvia Bordiga[2,], Jorge Gascon[1,*] and Unni Olsbye[3,*]*

[1]King Abdullah University of Science and Technology, KAUST Catalysis Center (KCC), Thuwal 23955, Saudi Arabia.

[2]Department of Chemistry, NIS Center and INSTM Reference Center, University of Turin, 10125, Turin, Italy.

[3]SMN Centre for Materials Science and Nanotechnology, Department of Chemistry, University of Oslo, N-0315 Oslo, Norway.

[4]The Smart Materials Research Institute, Southern Federal University, Sladkova 178/24, Rostov-on-Don, 344090, Russian Federation.

***Corresponding author**: unni.olsbye@kjemi.uio.no, jorge.gascon@kaust.edu.sa, silvia.bordiga@unito.it

Contents

S1. CO_2 hydrogenation thermodynamics S2
S2. Kinetic model for CO_2 hydrogenation S4
S3. Regeneration of the $PdZn/ZrO_2$+SAPO-34 multifunctional catalyst S11
S4. Chemical and textural characterization of catalysts S12

S5. Spectroscopic characterization of the PdZn/ZrO_2 .... S14

S6. Imaging of the PdZn/ZrO2 and PdZn/ZrO2+SAPO-34 catalysts…………...………...……S18

S1. CO_2 hydrogenation thermodynamics

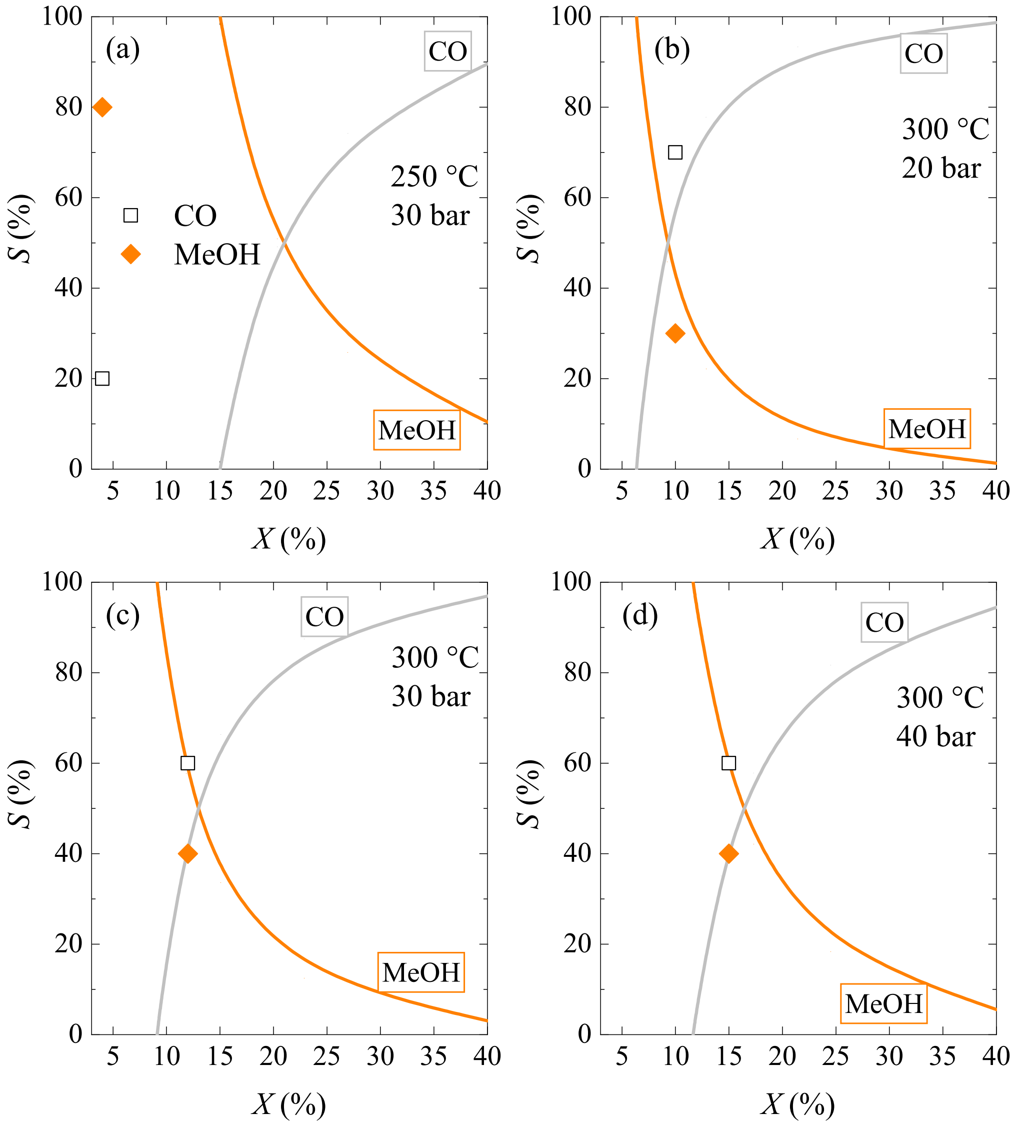

Figure S1. Thermodynamics of the CO_2/methanol equilibrium. The lines represent the CO_2/MeOH equilibrium selectivities and the dots the obtained experimental data for the PdZn/ZrO_2 catalyst

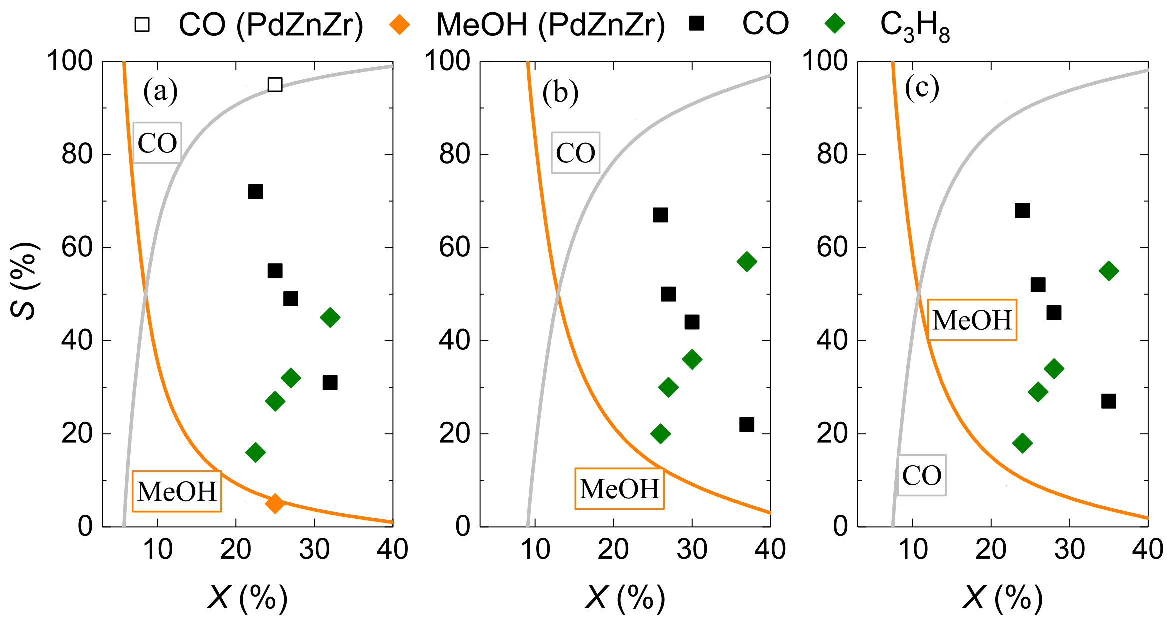

Figure S2. Thermodynamics of the CO_2/methanol equilibrium. The lines represent the CO_2/MeOH equilibrium selectivities and the dots the obtained experimental data for the PdZn/ZrO_2+SAPO-34 system. a) 30 bar, b) 40 bar, c) 50 bar. Open square and orange diamond in chart a) are the experimental CO and MeOH selectivity obtained at these conditions with the PdZn/ZrO_2 catalyst

S2. Kinetic model for CO_2 hydrogenation

The kinetic model for the conversion of CO_2 into methanol/propane was developed using the mass conservation equation of each component of the reaction medium. Several simplifications can be assumed due to the characteristics of the system. The catalyst activity remained almost constant during reaction. This avoids the need for a time-dependent expression of the equation and the intrinsic kinetic model can be extracted from these steady-state experiments. The reactor diameter is ca. 2 mm, which avoids radial dispersion and permits assuming a purely convective transport of the gas. The pressure in all channels was accurately controlled by a membrane system and temperature was maintained constant in each set of channels. Therefore, isothermal and isobaric conditions can also be assumed. In summary, the steady-state design equation for a packed bed reactor can be used. Then, for the molar fraction of each *i* component of the reaction medium:

$$\frac{F}{S}\frac{dy_i}{dl} = \rho r_i, \tag{S1}$$

defined for a catalytic bed length within $0 < l < L$, where F is the molar flow rate, S is the reactor cross section, ρ is the catalytic bed density and r_i is the formation rate of each *i* compound.

These formation rates are calculated from the reaction rate of each *j* individual reaction. In order to avoid mathematical uncertainty and strictly compare reaction rates two equilibrium reactions were considered: CO_2 hydrogenation to methanol and the reverse water-gas shift reaction[1]. When the acid SAPO-34 function was added to the catalytic system, a third reaction was considered: the conversion of methanol into propane. In this case a fast conversion of methanol to propane is assumed, with both methanol-to-propene and propene hydrogenation considered quite faster that CO_2/CO/MeOH equilibria. In this way, reaction network is defined by the following reactions:

$$CO_2 + 3H_2 \overset{1}{\rightleftarrows} CH_3OH + H_2O \tag{S2}$$

$$CO_2 + H_2 \overset{2}{\rightleftarrows} CO + H_2O \tag{S3}$$

$$3\,CH_3OH + H_2 \xrightarrow{3} C_3H_8 + 3\,H_2O \tag{S4}$$

Equilibrium constants for the two first reactions were considered with empirical correlations[2]. Although reactants/products adsorption could play a role and adsorption constants could have also been considered, decreasing the mathematical error encourages us to use potential expressions for the estimation of reaction rates, thereby minimizing the number of parameters to be fitted and maximizing the accuracy of the calculations. Then, kinetic equations are defined as:

$$r_1 = k_1 P_{CO_2} P_{H_2}^3 - \frac{P_{CH_3OH} P_{H_2O}}{K_1} \tag{S5}$$

$$r_2 = k_2 P_{CO_2} P_{H_2} - \frac{P_{CO} P_{H_2O}}{K_2} \tag{S6}$$

$$r_3 = k_3^{'} P_{CH_3OH} \tag{S7}$$

Please note that due to the low partial pressure of methanol and the H_2 surplus because of the low CO_2 conversion, reaction 3 was considered only dependent on methanol partial pressure. Then, k'_3 can be considered as an apparent rate constant. For the same reason, another simplification is to use the stoichiometric rate expression for the equilibrium-limited reaction of methanol formation (Eq. S5).

In each case, the system is solved using a Runge-Kutta method of orders 1-5, in order to optimize the reaction constants k_j. With this aim, an objective function based on the sum of square errors (*SSE*) between the calculated and experimental values of molar fractions (y_i and y_i^e, respectively) is defined,

$$SSE = \sum_{i=1}^{n_c} \omega_i \sum_{n=1}^{n_e} \left(y_i - y_i^e \right)_n^2 , \tag{S8}$$

where ω_i is the weight factor of each i compound and n_c and n_e are the number of compounds and experiments, respectively.

After the optimization of the kinetic parameters, the fitting of the experimental data obtained using both the stand-alone PdZn/ZrO_2 catalyst and the multifunctional PdZn/ZrO_2+SAPO-34 catalytic system with the constant in Table S1 is shown in Figure S3, whereas the estimated rates are illustrated in Figures S4-S5. Due to the different reaction orders, the rate constants cannot be directly compared. Instead, we focus on individual reaction rates versus contact time.

Table S1. Estimated kinetic parameters for CO_2 hydrogenation at different temperatures.

		**PdZn/ZrO_2[a]**		**PdZn/ZrO_2+SAPO-34[b]**
	**(250 ºC)**	**(300 ºC)**	**(350 ºC)**	**(350 ºC)**
k_1 (mol g^{-1} h^{-1} bar^{-4})	2.75 10^{-8}	6.58 10^{-8}	4.93 10^{-7}	7.05 10^{-6}
k_2 (mol g^{-1} h^{-1} bar^{-2})	8.22 10^{-6}	4.03 10^{-5}	1.32 10^{-4}	1.6 10^{-4}
k'_3 (mol g^{-1} h^{-1} bar^{-1})				3.85 10^{-2}

[a]Data collected at 30 bar. [b]Data collected at 30, 40 and 50 bar

References

1. Sreetama Ghosh, Joby Sebastian, Louise Olsson, Derek Creaser. Experimental and kinetic modeling studies of methanol synthesis from CO2 hydrogenation using In2O3 catalyst, Chemical Engineering Journal, 416, 2021, 129120
2. Andrés T. Aguayo, Javier Ereña, Diana Mier, José M. Arandes, Martin Olazar, and Javier Bilbao. Kinetic Modeling of Dimethyl Ether Synthesis in a Single Step on a CuO−ZnO−Al2O3/γ-Al2O3 Catalyst, Industrial & Engineering Chemistry Research, 46, 2007, 5522–5530

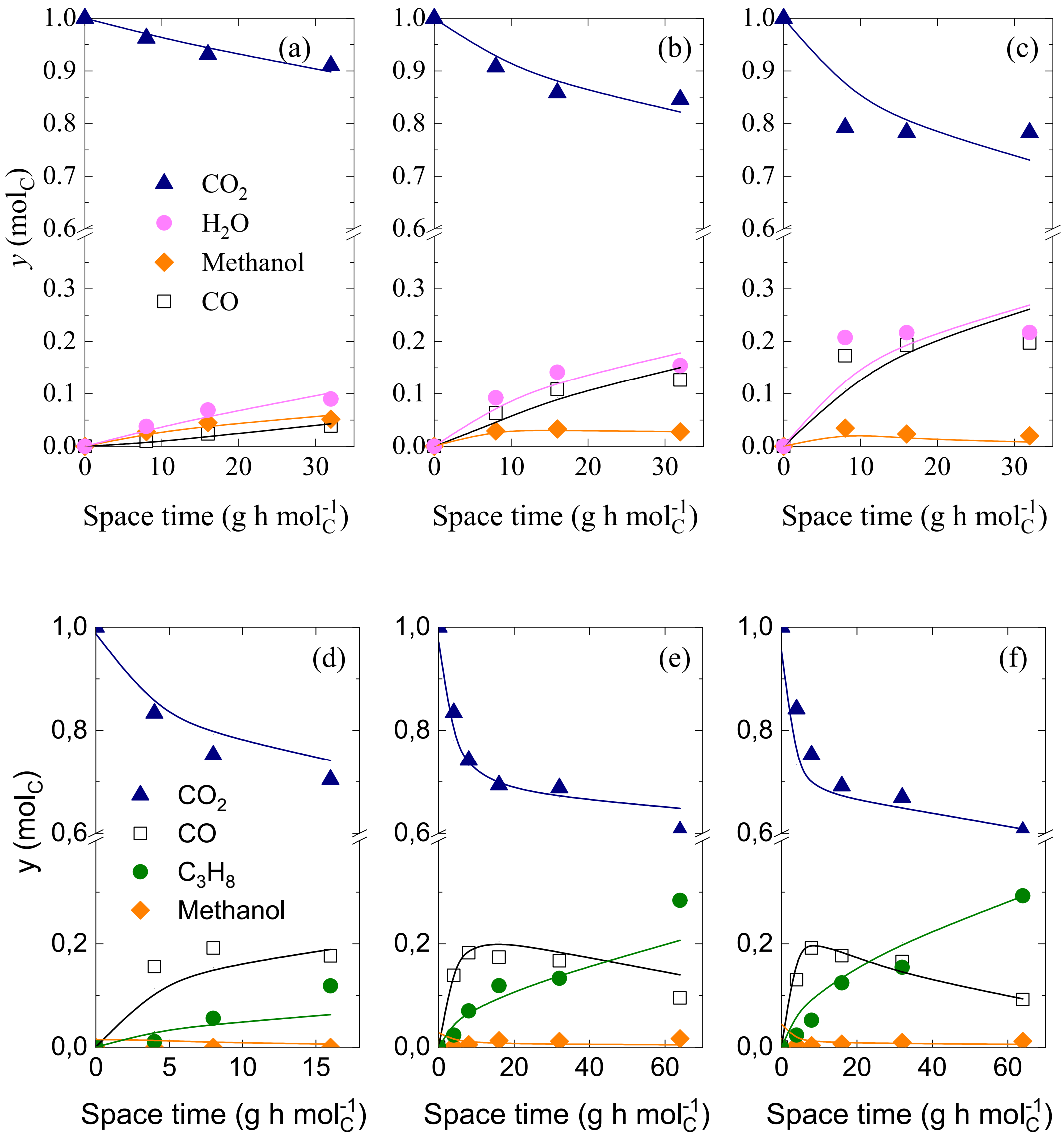

Figure S3. Experimental data fitting CO_2 to methanol over the PdZn/ZrO_2 catalyst at **a)** 250, **b)** 300 and **c)** 350 ºC and 30 bar. Experimental data fitting CO_2 to propane over the PdZn/ZrO_2+SAPO-34 system at 350 ºC **d)** 30, **e)** 40 and **f)** 50 bar.

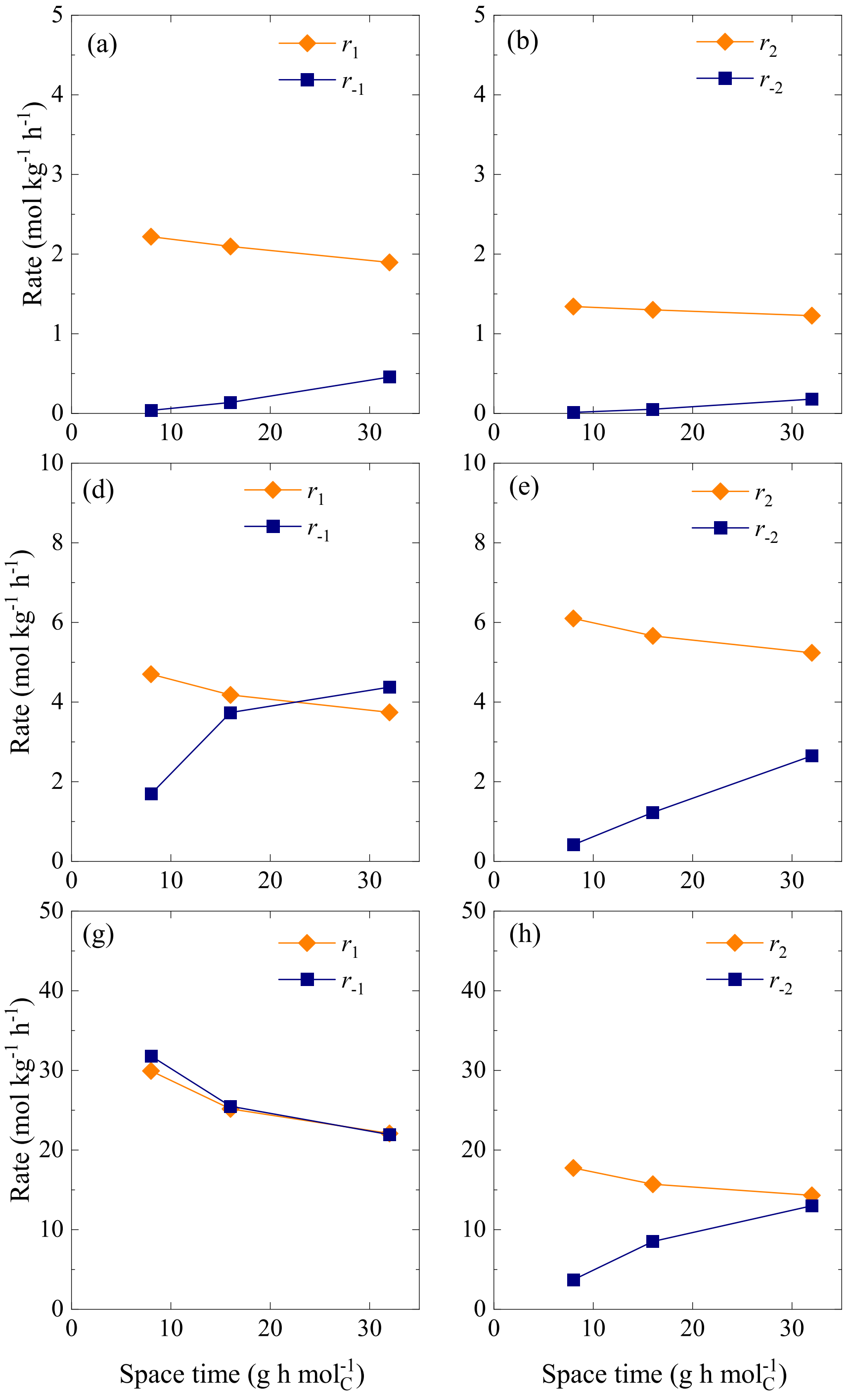

Figure S4. Reaction rates of CO_2 to methanol over the PdZn/ZrO_2 at (a, b) 250, (c, d) 300 and (e, f) 350 ºC and 30 bar. r_1, CO_2 hydrogenation (Eq. S2); r_2, rWGSR (Eq. S3).

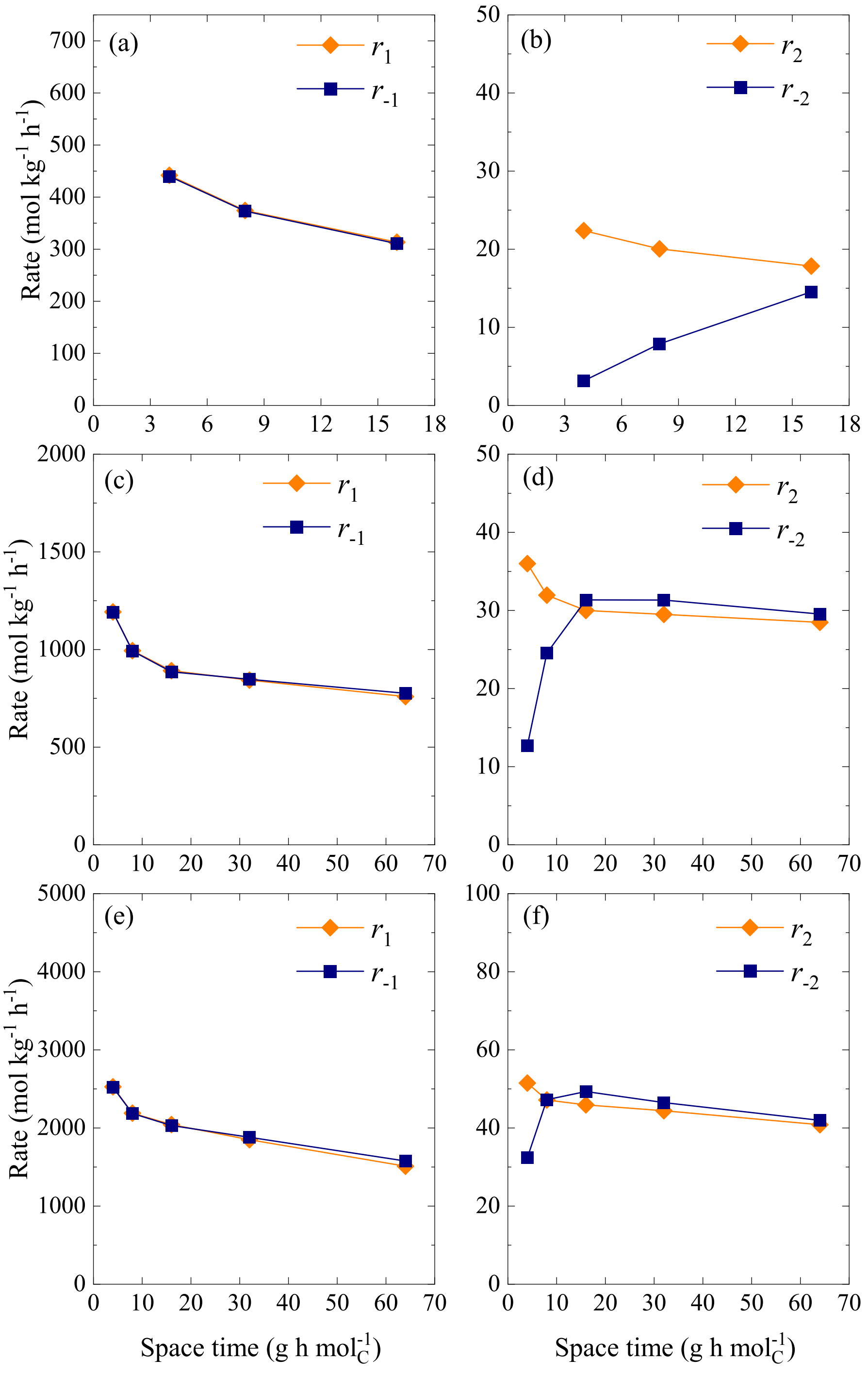

Figure S5. Reaction rates of CO_2 to propane over the PdZn/ZrO_2+SAPO-34 at 350 ºC and (a, b) 30, (c, d) 40 and (e, f) 50 bar. r_1, CO_2 hydrogenation (Eq. S2); r_2, rWGSR (Eq. S3) (the corresponding r_3, MeOH-to-propane (Eq. S4), is shown in Figure 3).

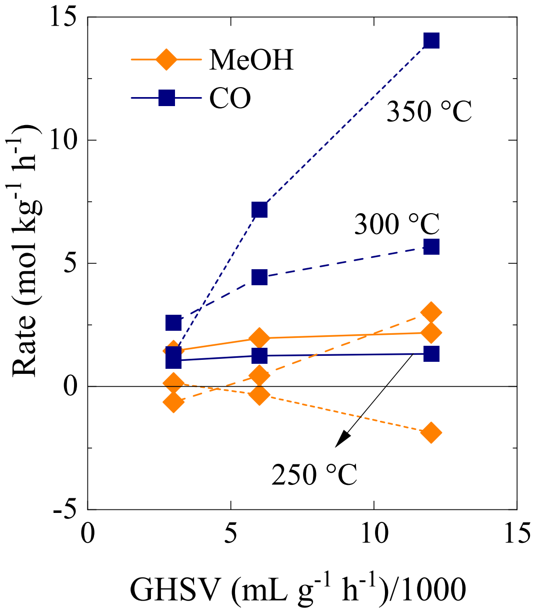

Figure S6. MeOH and CO net formation rates during CO_2 to methanol overt the PdZn/ZrO_2 at 30 bar.

S3. Regeneration of the PdZn/ZrO_2+SAPO-34 multifunctional catalyst

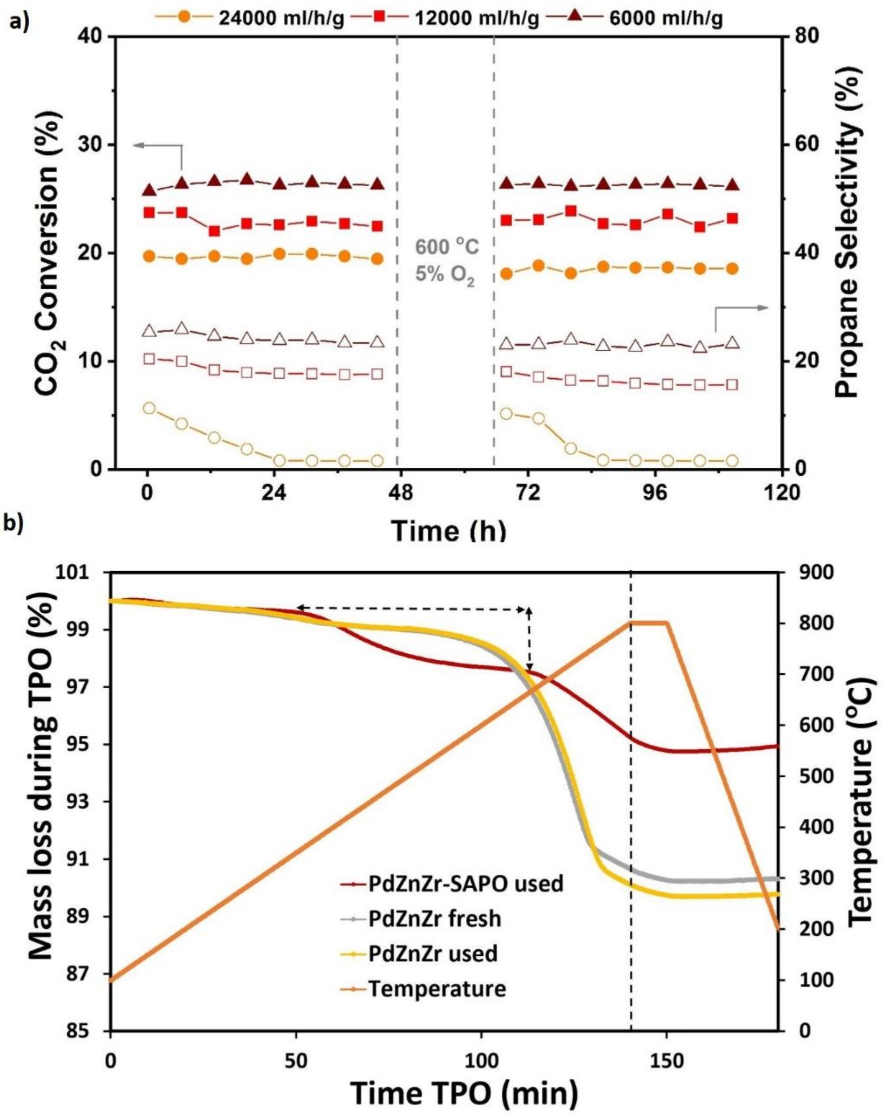

Figure S7. a) Catalytic performance of PdZn/ZrO_2+SAPO-34 combined system before and after regeneration at 600 ºC for the CO_2 conversion to hydrocarbons at different space times. CO_2:H_2 1:3, 350 ºC, 30 bar; b) TGA analysis of the spent hybrid system after 48 hours of reaction at 350 °C, 30 bar and 24000 ml/h/g. Both fresh and spent PdZn/ZrO_2 were also analyzed as reference.

S4. Chemical and textural characterization of catalysts

Table S2. Energy-dispersive X-ray spectroscopy (EDS) estimated composition of the $PdZn/ZrO_2$ sample (mol %).

Pd	Zn	Zr
2 ± 1	13± 3	85 ± 2

Table S3. Textural properties of SAPO-34 and ZSM-5 zeolites.

Zeolite	S_{BET} (m^2/g)	$S_{meso/ext}$ (m^2/g)	S_{micro} (m^2/g)	V_{total} (ml/g)	V_{micro} (ml/g)	NH_3 desorb/mass (mmol/g)
ZSM-5	417	120	296	0.23	0.12	0.57
SAPO-34	770	8	762	0.28	0.27	0.66

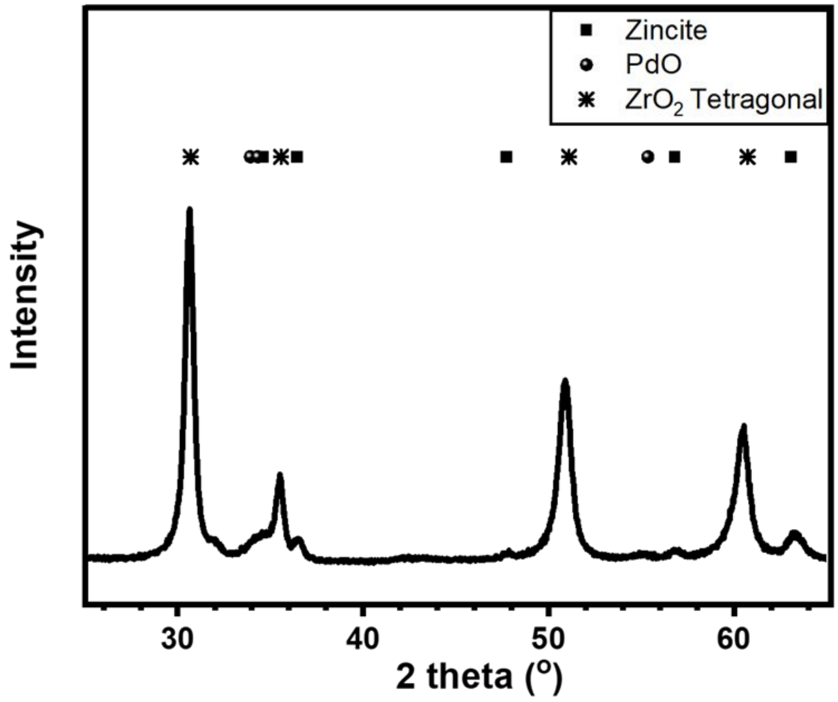

Figure S8. Powder X-ray diffraction (PXRD) pattern of the as prepared $PdZn/ZrO_2$ sample.

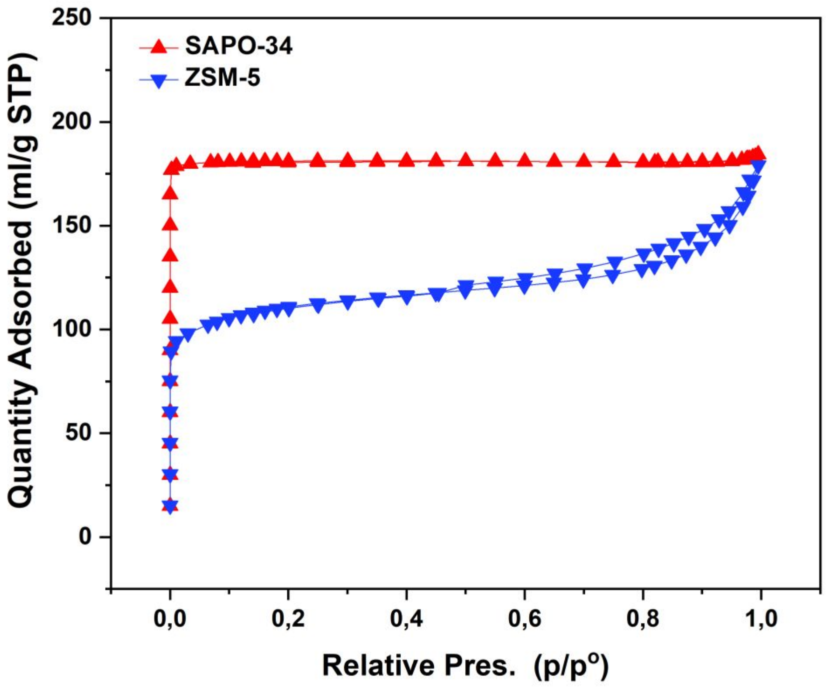

Figure S9. N_2 adsorption isotherms of SAPO-34 (red) and ZSM-5 (blue) zeolites.

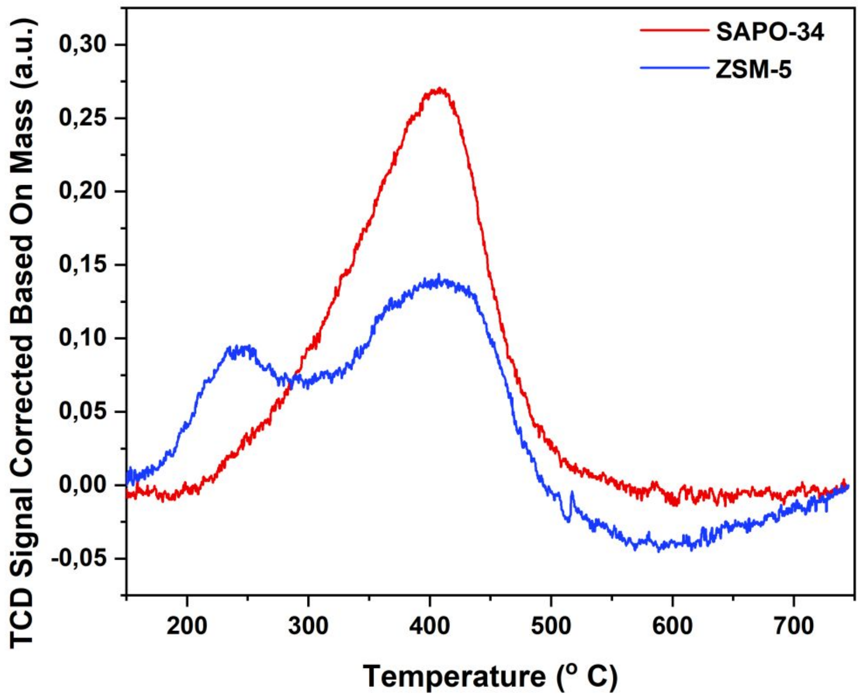

Figure S10. NH_3-TPD profiles of SAPO-34 (red) and ZSM-5 (blue) zeolites.

S5. Spectroscopic characterization of the $PdZn/ZrO_2$

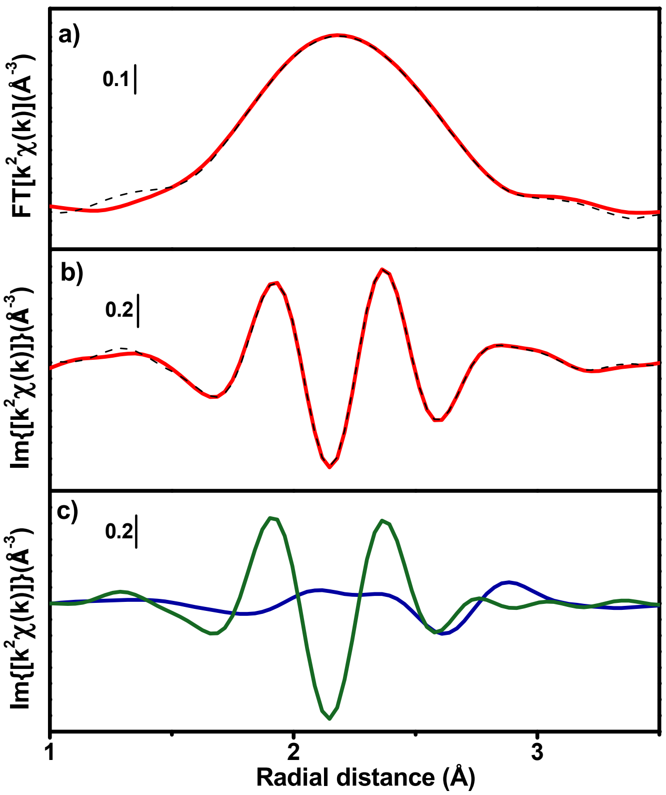

Figure S11. Experimental (red line) and best fit (dashed black line) of a) magnitude and b) imaginary parts of the k^2-weighted, phase-uncorrected FT-EXAFS spectra at Pd K-edge measured at 400°C/H_2. c) Imaginary part of Pd-Zn (green line) and Pd-Pd (blue line) scattering paths generated by FEFF6 resulting from the fit.

Table S4. Energy shift (E_0), radial distances (ΔR) and Debye Waller factors (σ^2) determined by EXAFS fit at the Pd K-edge. Fit was performed in R-space, in the range 1-3.5 Å, on FT-EXAFS spectra transformed in the 2-10.5 $Å^{-1}$ k-range, resulting in a number of independent parameters $\pi\Delta R\Delta k/2>9$. The passive amplitude reduction factor of 0.84 ± 0.03 was evaluated from EXAFS analysis of a reference Pd metal foil and set in the fit. As particles of dimension >10 nm was observed by microscopies measurements (see Figure S18), Pd-Zn and Pd-Pd coordination numbers were fixed at the bulk conditions of 8 and 4, respectively.

	**β1-PdZn**
**Fit R-factor**	0.002
**Npar (Nind)**	5(9)
**E_0 (eV)**	-5 ± 1
**$R_{Pd\text{-}Zn}$ (Å)**	2.610 ± 0.008
**$\sigma^2_{Pd\text{-}Zn}$ ($Å^2$)**	0.0149 ± 0.0003
**$R_{Pd\text{-}Pd}$ (Å)**	2.917 ± 0.017
**σ^2Pd-Pd ($Å^2$)**	0.022 ± 0.002

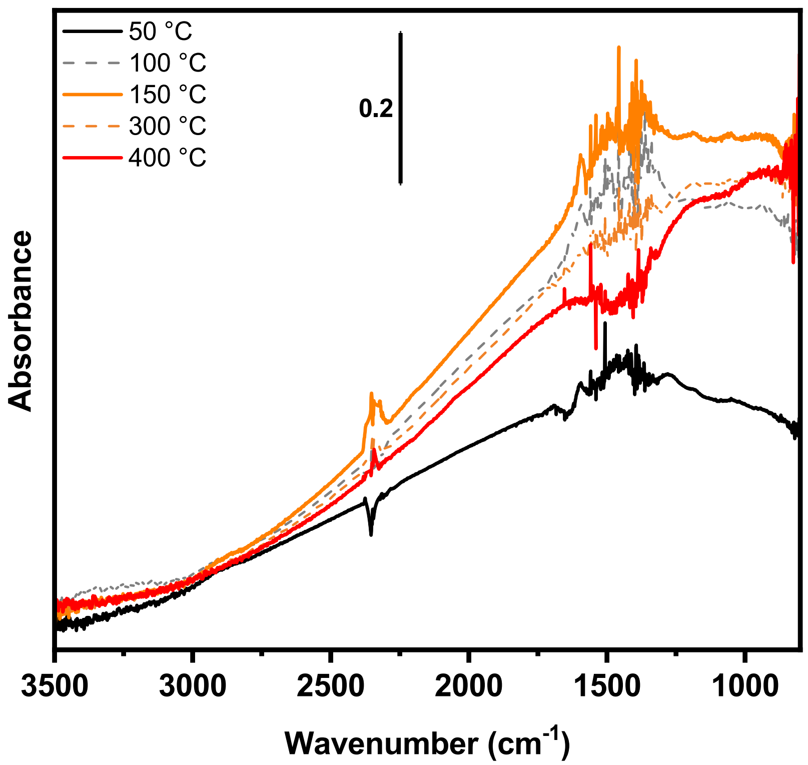

Figure S12. FT-IR difference spectra of $PdZn/ZrO_2$ catalyst in H_2 at increasing temperature (subtrahend spectrum is that recorded in oxygen at each same temperature).

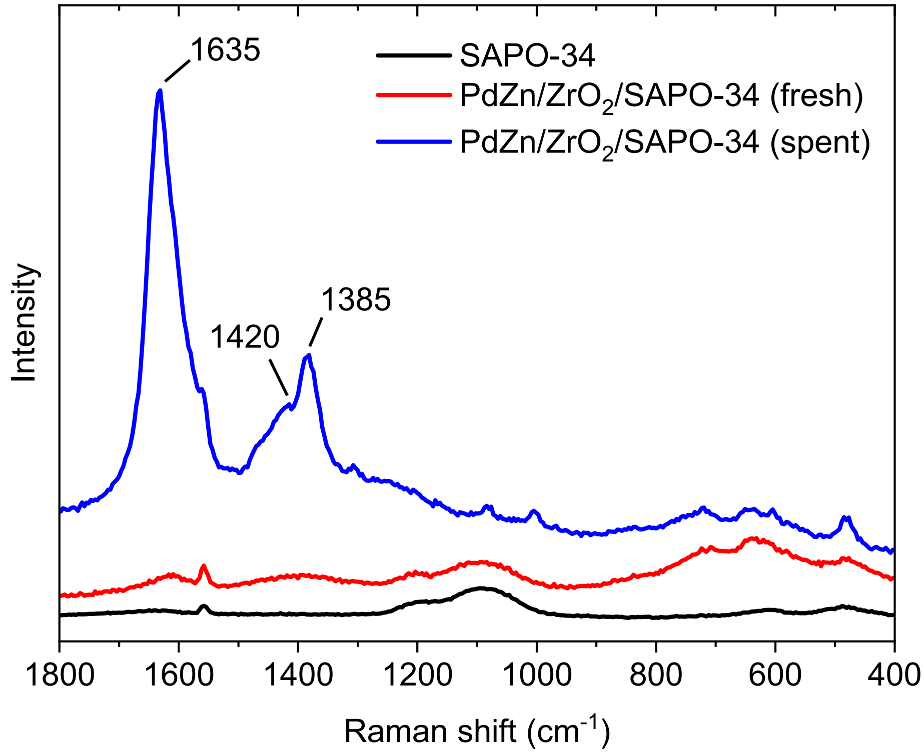

Figure S13. Raman spectra of SAPO-34 alone and fresh and spent PdZn/ZrO_2+SAPO-34 after 48 hours of reaction at CO_2:H_2 1:3, 350 ºC, 30 bar and 24000 GHSV.

S6. Imaging of the PdZn/ZrO$_2$ and PdZn/ZrO2+SAPO-34 systems.

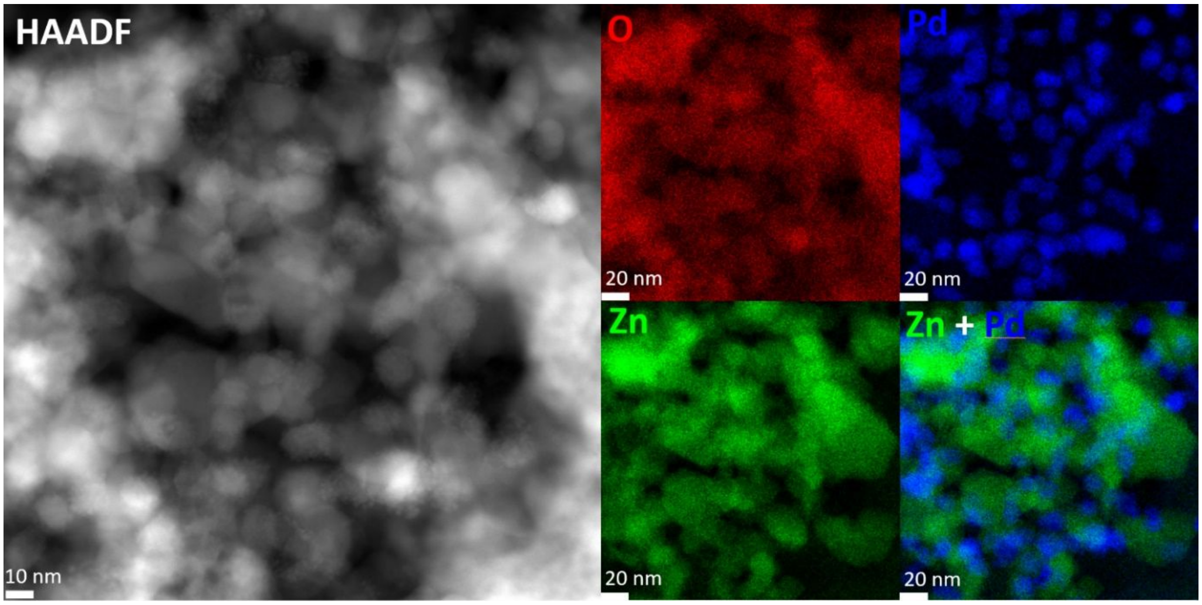

Figure S14. Low magnification HAADF-STEM imaging of as-synthesized PdZn/ZrO$_2$ catalysts and related elemental maps built with Kα emission lines provided by Pd, O, and Zn atoms.

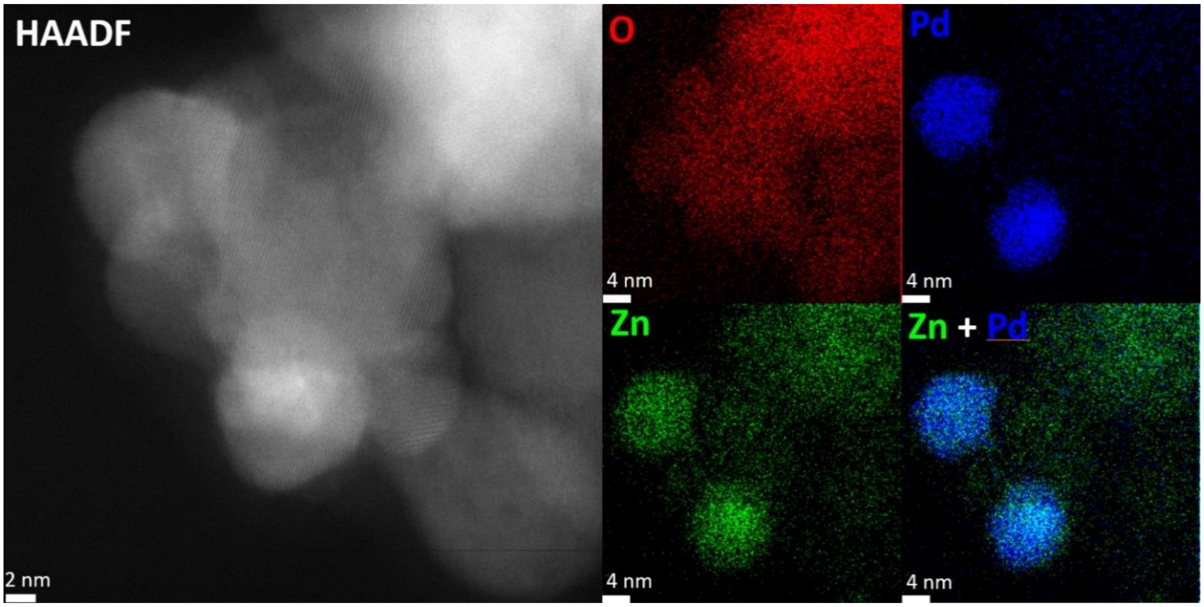

Figure S15. High-resolution HAADF-STEM imaging of a PdZn alloy nanoparticle after H_2 activation handled without air exposure and related elemental maps built with Kα emission lines provided by Pd, O, and Zn atoms.

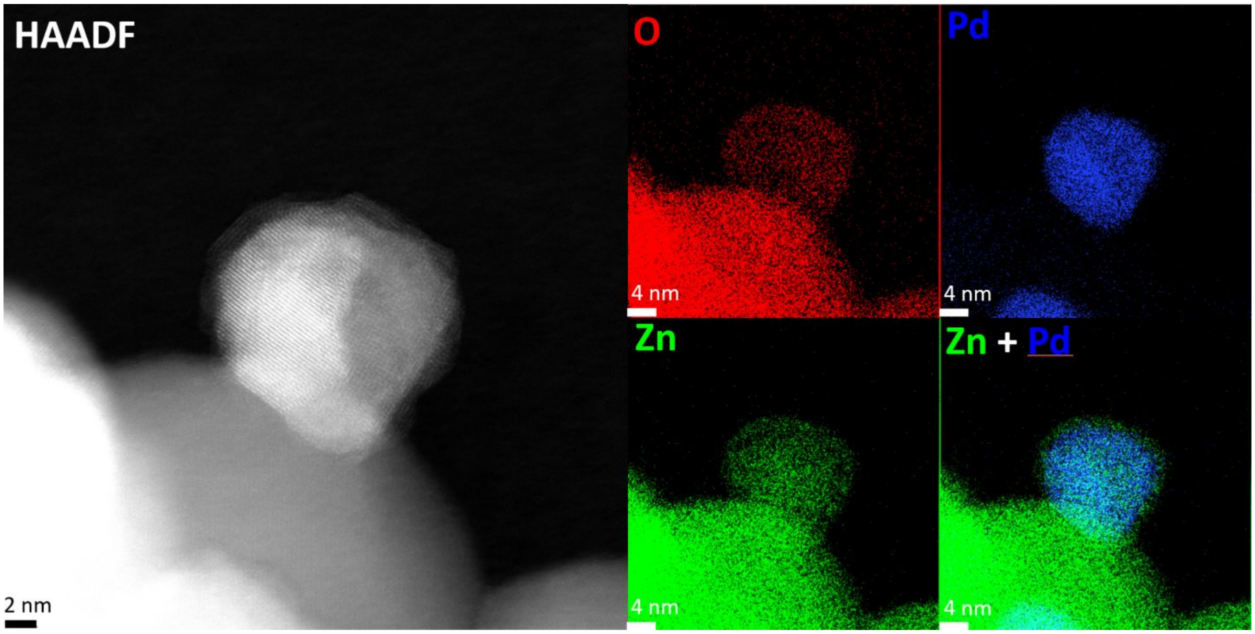

Figure S16. High-resolution HAADF-STEM imaging of a PdZn alloy nanoparticle after CO_2 hydrogenation handled without air exposure and related elemental maps built with Kα emission lines provided by Pd, O, and Zn atoms.

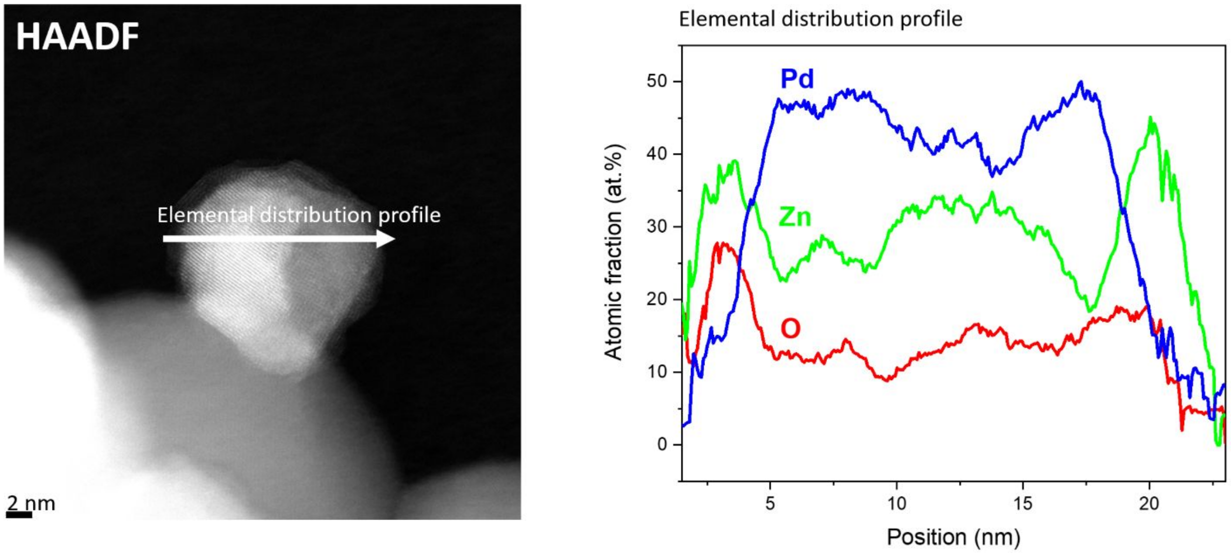

Figure S17. Elemental distribution profile calculated from STEM-EDS data of a PdZn alloy nanoparticle after CO_2 hydrogenation handled without air exposure.

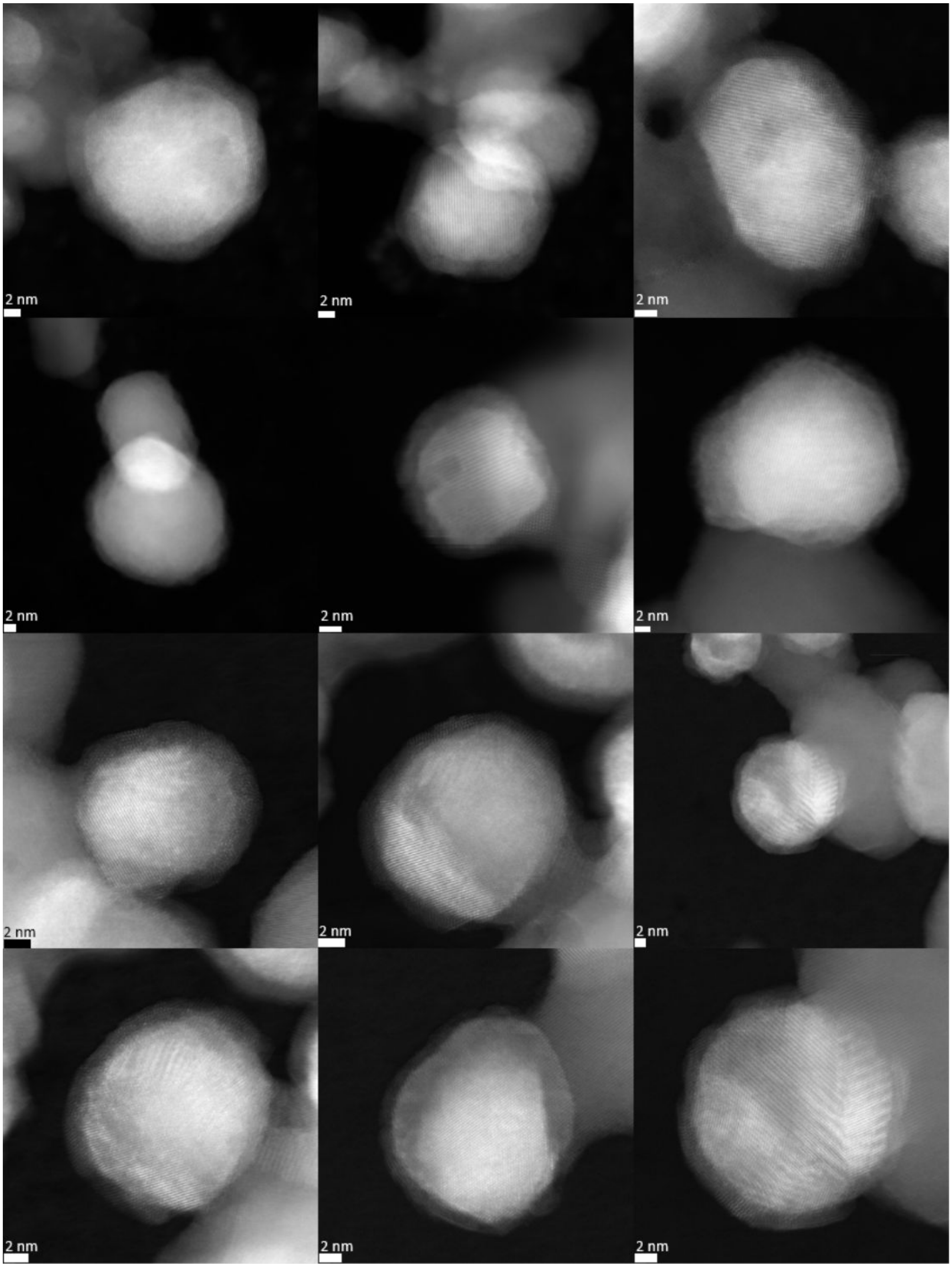

Figure S18. High-resolution HAADF-STEM imaging of the PdZn alloy after CO_2 hydrogenation.
